# Detection of urothelial carcinoma in Lynch syndrome using microsatellite instability analysis of urine cell-free DNA

**DOI:** 10.1016/j.ebiom.2025.105969

**Published:** 2025-10-25

**Authors:** Rebecca Hall, Richard Gallon, Christine Hayes, Patricia Herrero-Belmonte, Rachel Phelps, Donna Job, Ruth Wake, Mary Ferrier, Helen Turner, Rachel O'Donnell, Arjun Nambiar, Bhavan Rai, Richard Martin, Ciaron McAnulty, Mauro Santibanez-Koref, Rakesh Heer, Michael S. Jackson, John Burn

**Affiliations:** a Translational and Clinical Research Institute, Faculty of Medical Sciences, Newcastle University, Newcastle upon Tyne, NE2 4HH, UK; b The Newcastle upon Tyne Hospitals NHS Foundation Trust, Newcastle upon Tyne, NE7 7DN, UK; c Biosciences Institute, Faculty of Medical Sciences, Newcastle University, Newcastle upon Tyne, NE2 4HH, UK; d Division of Surgery, Imperial College London, London, SW7 2AZ, UK

**Keywords:** Lynch syndrome, Urothelial carcinoma, Urinary tract cancer, Screening, Microsatellite instability, Mismatch repair deficiency, Urine cell-free DNA, Liquid biopsy

## Abstract

**Background:**

Urothelial carcinoma is the third most common cancer in Lynch syndrome but there is no approved screening method. Lynch syndrome cancers are characterised by high levels of microsatellite instability (MSI/MSI-H). Here, we assess the feasibility of urine MSI analysis for non-invasive urothelial carcinoma screening.

**Methods:**

We analysed urine cell-free DNA samples from two cohorts using an amplicon-sequencing MSI assay: (1) Sequential cases of upper tract urothelial carcinoma (UTUC) provided paired tumour/pre-operative urine samples for MSI analysis and mismatch repair protein immunohistochemistry (MMR IHC) and those with an MMR deficient (MMRd) tumour were offered constitutional Lynch syndrome testing (2) Eligible individuals with a diagnosis of *MSH2*-Lynch syndrome (aged 30–75 years, without recent cancer diagnosis) were offered urine MSI analysis via a clinic appointment or postal urine sample collection. All cases were followed up for at least 12 months.

**Findings:**

Three of 50 cases of UTUC (6.00%) were MSI-H, MMRd by IHC and detectable by MSI-H or borderline-MSI-H urine signals. All occurred in individuals with new Lynch syndrome diagnoses. Urines and tumours from the remaining 47 patients were microsatellite stable (MSS) and tumours were MMR proficient by IHC. Urine MSI analysis achieved 100% sensitivity and specificity for symptomatic UTUC (95% CI 29.2%–100% and 92.4%–100% respectively). 81 of 142 eligible individuals with a diagnosis of *MSH2*-Lynch syndrome participated (57.0%) with one later excluded following urothelial carcinoma diagnosis between enrolment and sample collection. MSI-H urines were identified in 5/80 (6.25%), with urothelial carcinoma subsequently diagnosed in 4/5. None of the 75 participants with an MSS urine had a urothelial carcinoma diagnosis during follow up. Urine MSI analysis achieved 100% sensitivity, 98.7% specificity, 80% positive predictive value, and 100% negative predictive value for asymptomatic Lynch syndrome urothelial carcinoma (95% CIs: 39.8–100%, 92.9–99.9%, 28.4–99.5% and 95.2–100% respectively).

**Interpretation:**

Urine MSI testing using a low-cost assay which is suitable for routine surveillance can detect asymptomatic Lynch syndrome urothelial carcinoma, facilitating early diagnosis. Further studies are needed to define accuracy and patient outcomes.

**Funding:**

10.13039/501100003776Newcastle upon Tyne Hospitals NHS Charity/The Sir Bobby Robson Foundation, The 10.13039/501100007568Urology Foundation, The 10.13039/100032252Barbour Foundation, 10.13039/501100000289Cancer Research UK.

**Research in context**

**Evidence before this study**

A search of the PubMed database was performed using the following key words individually or in combination: ‘Lynch syndrome’, ‘Urinary tract cancer’, ‘urothelial carcinoma’, ‘surveillance’, ‘screening’, ‘microsatellite instability’. We reviewed any peer-reviewed, original articles, as well as review articles by bodies relevant to UK/European clinical guidance in Urology and/or Clinical Genetics published prior to 01/07/2025 (no language restriction). Individual studies assessing urine cytology (2008) and urinalysis-based screening (2022) in Lynch syndrome cohorts reported sensitivities of 29% (n = 977) and 0% (n = 204) respectively. A 2022 review by the European Association of Urology included a recommendation regarding upper tract urothelial carcinoma screening for individuals with a Lynch syndrome diagnosis using a combination of urinalysis, urine cytology and imaging (ultrasound or CT depending on risk category) from age 45–50 years. However, a review/practice guideline by the European Hereditary Tumour Group (2021) did not recommend urinary tract cancer screening based on the lack of demonstrated benefit of surveillance. The search did not identify any studies exploring the use of urine microsatellite instability (MSI) testing in Lynch syndrome cohorts.

A further search of PubMed, using the search terms ‘urine’, ‘liquid biopsy’, ‘cell-free DNA’, ‘urothelial carcinoma’, ‘bladder’, and ‘upper tract’ reveals an extensive field of research exploring a wide range of different liquid biopsy approaches for the detection of urothelial carcinoma in non-Lynch syndrome populations with promising results, summarised in several extensive review articles. When ‘microsatellite instability’ is added, the only report of note is our proof of principle study where urine MSI analysis detected a symptomatic upper tract urothelial carcinoma in an individual with a diagnosis of Lynch syndrome. Other search results describe analysis of polymorphic microsatellites to detect copy number variants in urine from patients with a diagnosis of symptomatic bladder cancer, an approach which does not directly compare to our methodology using MSI analysis to detect mismatch-repair deficient urothelial carcinoma in an asymptomatic Lynch syndrome cohort.

**Added value of this study**

This study explores the use of urine MSI analysis-based screening for urothelial carcinoma in the high-risk Lynch syndrome population. Urine MSI analysis of 80 individuals with a diagnosis of *MSH2*-Lynch syndrome recruited from Northeast England identified four asymptomatic urothelial carcinomas, including two low-grade, early stage (Ta) upper tract urothelial carcinomas which were treatable endoscopically. In comparison to previous work exploring urinalysis or urine cytology-based screening, urine MSI analysis demonstrated high sensitivity and specificity after a minimum 12 months follow up of each participant.

**Implications of all the available evidence**

Urine MSI analysis may provide a much-needed screening test for urothelial cancer in Lynch syndrome. Further study of larger and longitudinal cohorts is required to assess the impact of screening on morbidity and mortality. Additionally, assessments of patient acceptability and cost-effectiveness will be needed. The low cost and high-throughput MSI assay used here is already in routine clinical use for colorectal cancer testing, which will facilitate clinical adoption.

## Introduction

Lynch syndrome is a cancer predisposition syndrome affecting approximately 1 in 300 people.^1^ There are four sub-types resulting from constitutional pathogenic variants in one of four DNA mismatch repair (MMR) genes: *MLH1*, *MSH2*, *MSH6* and *PMS2.* Lynch syndrome neoplasia acquire MMR deficiency (MMRd) following a somatic second hit.^2^ Microsatellite instability (MSI) is a molecular phenotype of MMRd resulting from failure to repair DNA polymerase slippage in short tandem repeats and its presence in bodily fluids can indicate the presence of an MMRd cancer.^3^^,^^4^

Urothelial carcinoma is the second or third commonest Lynch syndrome cancer after colorectal cancer (CRC) and endometrial cancer. 5 The Prospective Lynch Syndrome Database (PLSD) reports that 28.5% of individuals with a diagnosis of *MSH2*-Lynch syndrome develop urothelial carcinoma during their lifetime, in comparison to 10% and 8% of individuals with a diagnosis of *MLH1-* and *MSH6*-Lynch syndrome, respectively.^5^ Upper tract urothelial carcinoma (UTUC), arising in the renal pelvis or ureter, is particularly common in Lynch syndrome with an estimated 14-fold increased risk compared to the general population.^6^ Notably, around 6% of all patients with UTUC have Lynch syndrome.^7^

The UK has national guidance regarding colonoscopy, aspirin prophylaxis and risk-reducing hysterectomy to manage Lynch syndrome cancer risk.^8^ For urothelial carcinoma, the European Association of Urology has suggested a screening protocol from the age of 45 years, using urinalysis, urine cytology and abdominal ultrasound/CT imaging.^6^ However, urinalysis for haematuria and urine cytology have poor sensitivity and specificity^9^^,^^10^ and the European Hereditary Tumour Group and the UK Cancer Genetics Group do not recommend Lynch syndrome urothelial carcinoma screening due to the lack of supportive evidence.^1^^,^^8^ With no effective surveillance, urothelial carcinoma may account for up to 5% of *MSH2*-Lynch syndrome mortality.^11^

We present urine cell-free DNA (ucfDNA) based detection of MMRd urothelial carcinoma using the Newcastle MSI-Plus assay, a low cost and scalable MSI and mutation hotspot test accredited in the UK for diagnostic use on CRC biopsies that can produce reliable results from <1 ng of sample DNA.^12^ In a regional pilot study aiming to demonstrate the feasibility of using the assay as a Lynch syndrome urothelial carcinoma screening test, we screened urine samples from asymptomatic individuals with a diagnosis of *MSH2*-Lynch syndrome and unselected cases of UTUC to calculate initial estimates of the sensitivity and specificity of the assay for MMRd urothelial carcinoma detection.

## Methods

### Project design and participant identification

**Fig. 1:**
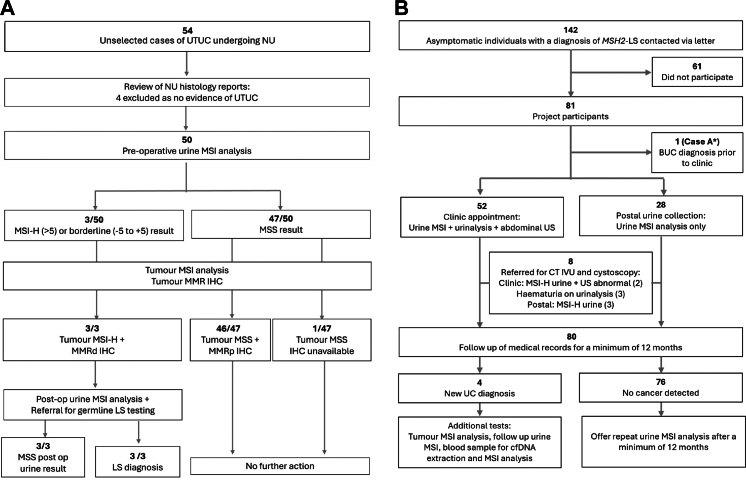
**Workflows for the unselected UTUC cohort (A) and *MSH2*-Lynch syndrome cohort (B).** Following exclusions, the sample sizes for cohorts A and B were 50 and 81 respectively. Abbreviations used: **BUC**, bladder urothelial carcinoma; **cfDNA**, cell free DNA; **CT IVU**, computerised tomography intravenous urogram; **LS**, Lynch syndrome; **MSI**, microsatellite instability; **MSI-H**, high levels of microsatellite instability; **MMR IHC**, MisMatch Repair proteins ImmunoHistoChemistry; **MMRd**, mismatch repair deficiency; **MMRp**, mismatch repair proficiency; **MSS**, microsatellite stable; **NU**, nephroureterectomy; **UC**, urothelial carcinoma; **US**, ultrasound; **UTUC**, upper tract urothelial carcinoma; **LS**, Lynch syndrome.

Samples were collected prospectively from two cohorts and analysed following the workflows shown in Fig. 1. Cohort 1 consisted of patients with known diagnoses of UTUC to assess urine MSI analysis for detection of symptomatic disease, whilst cohort 2 consisted of asymptomatic individuals with a diagnosis of *MSH2*-Lynch syndrome. We focussed upon *MSH2*-Lynch syndrome, rather than individuals with *MLH1*, *MSH6* or *PMS2* variants, as this is the population most in need of urothelial carcinoma surveillance and to increase the likelihood of detecting asymptomatic disease. We offered equal opportunity for project participation to both sexes, with all eligible individuals approached in the same manner for cohorts 1 and 2. Sex data were collected from the National Health Service (NHS) clinical databases (self-reported by participants) for the Newcastle upon Tyne Hospitals NHS Foundation Trust and the regional Clinical genetics department.

### Cohort 1: unselected UTUCs to assess detection of symptomatic MMRd tumours

Pre-operative urines and matched tumour were collected from consecutive consenting patients admitted to our tertiary urology centre for nephroureterectomy from 1/12/22 to 1/10/24 within The Newcastle upon Tyne Hospitals NHS Foundation Trust. Patients were included in the study if there was evidence of UTUC at histology of the nephroureterectomy specimen (see the Statistics section for further details of study design and eligibility for cohort 1). If pre-operative urine or tumour analyses were microsatellite instability-high (MSI-H), a post-operative urine sample was collected for MSI analysis. Tumour mismatch repair protein immunohistochemistry (MMR IHC) was performed for all cases. Participants with an MMRd tumour were offered constitutional genetic testing for Lynch syndrome using the standard NHS pathway via the Northeast and Yorkshire Genomic Medicine Service.^13^

### Cohort 2: individuals with a diagnosis of *MSH2*-Lynch syndrome to assess detection of asymptomatic MMRd tumours

Individuals aged 30–75 years with a likely pathogenic/pathogenic *MSH2* variant and without a cancer diagnosis in the past 12 months were identified in April 2022, using our regional NHS database covering Northeast England/North Cumbria. We identified 142 individuals eligible to participate and, based on a predicted ∼50% uptake into the project and the urothelial carcinoma incidence rates reported by the PLSD,^5^ we anticipated that one or more individuals would have asymptomatic UC. This would provide the opportunity to demonstrate urine MSI analysis can detect asymptomatic disease, which would be novel and a significant foundation for future studies in larger populations (see the Statistics section for further details of study design and eligibility for cohort 2). Those aged 40–75 years were offered review in a joint Genetics/Urology clinic between December 2022 and June 2023. Participants attending clinic underwent a symptom review and were offered urinalysis for haematuria, abdominal ultrasound (unless urinary tract imaging had already been performed within 6 months) and urine MSI analysis. Urinalysis and MSI analysis used the same sample. Abdominal ultrasound was performed by an NHS practitioner unaware of the urine MSI result. For those aged 30–39 years, and those who declined travel to clinic, we offered postal urine sample collection for MSI analysis only.

Individuals with an MSI-H/borderline urine result (see below) or 1+ haematuria on two separate samples (without evidence of a urinary tract infection) were investigated according to the local pathway for unexplained haematuria using Computed Tomography Intravenous Urography (CT-IVU) and flexible cystoscopy. If an individual with an MSI-H urine result had normal urological investigations and endometrial cancer was a possibility, a gynaecology urgent referral pathway was available given we have previously observed MSI-H urine in a patient with a diagnosis of endometrial cancer associated with Lynch syndrome.^4^

For participants found to have urothelial carcinoma, we performed paired tumour MSI analysis and repeated urine collections at the time of tumour resection and urology follow-up appointments. Urine cytology was not performed routinely but we reviewed the results for cases where it was requested by the urology team. We reviewed the red blood cell count (RBCC) of the urine microscopy performed prior to ureterorenoscopy (URS) or nephroureterectomy for patients whose urothelial carcinoma diagnosis was made following postal urine sample collection. A blood sample was taken from patients with an MSI-H urine result for plasma cell-free DNA MSI analysis.

Twelve months after completion of the first phase of urine sample collections for MSI analysis, we began contacting all participants for whom a cancer was not detected via the project to confirm the patient had not received a diagnosis of urothelial carcinoma or endometrial cancer via any other route of investigation and offered repeat postal urine MSI testing.

### Sample collection

Urine samples were collected using the Colli-Pee® system including DNA preservative (DNA Genotek Inc). For participants of cohort 1 (unselected UTUC), pre-operative samples were collected on admission (usually 07:30–08:00). Post-operative samples were collected at least 24 h following surgery. Participants of cohort 2 (asymptomatic *MSH2*-Lynch syndrome) were asked to collect a first void morning sample (to bring to clinic or send in the post). We obtained experimental confirmation from the manufacturer that the DNA preservative did not affect urinalysis for haematuria. UcfDNA was extracted from 6 ml of urine using the QIAamp Circulating Nucleic Acid Kit (Qiagen 55114).The protocol was adjusted to use of 6 ml rather than 4 ml of urine and concentrations and volumes of assay components altered accordingly.

Tumour resection or biopsy tissue was collected as 3 × 10 μm formalin-fixed paraffin-embedded (FFPE) curls with histopathologist confirmation of ≥10% tumour content. Tumour DNA was extracted using the Maxwell RSC DNA FFPE Kit (Promega AS1720).

### MSI analysis

MSI analysis used the existing pathway for tumour testing at the regional NHS Genomics Laboratory Hub, the Newcastle upon Tyne Hospitals NHS Foundation Trust. This uses the Newcastle MSI-Plus assay, a multiplex PCR and amplicon sequencing-based assay of 14 MSI markers for high-throughput, automated MSI analysis, as previously described.^14^ The assay was clinically validated for CRC MSI analysis in 2022 and has UKCA accreditation for this purpose. Sequence analysis to generate sample MSI scores is automated using the MSI App described in detail in previous publications,^12^^,^^14^ supplied to laboratory teams using the assay. In brief, an MSI score is calculated using a naïve Bayesian classifier trained on sequence data from CRCs of known MSI status, supported by quality control metrics. The probability of instability is calculated for each marker based on the frequency of variant alleles and the individual ratios are multiplied together on a logarithmic scale. MSI scores range from approximately −25 to +30, with scores >0 or <0 indicating the sample is MSI-H or microsatellite stable (MSS), respectively.

According to the current NHS diagnostic protocol, MSI scores from −5 to +5 are considered ‘borderline’ and trigger repeat testing of CRC tumours to confirm the result. The −5 and +5 thresholds represent a 100,000-fold relative probability of being MSS vs MSI-H or MSI-H vs MSS respectively—results outside of this region are unlikely to cause a misclassification and the repeat testing burden of ∼2% caused by this range was deemed manageable. Repeat sample collection and analysis were therefore performed for ucfDNA or urothelial carcinoma tumour samples with results within this range. Given that ucfDNA may have a lower tumour content than tumour tissue, that the classifier was trained on CRCs which have more easily detectable and longer microsatellite insertion-deletion variants than in some other tumour types, and that the assay was being used as a screening test, borderline results (which could not be repeated or remained in the borderline range following repeat testing) were treated equivalent to MSI-H results for Lynch syndrome urothelial carcinoma surveillance.^15^ MMR IHC results were not available to the NHS clinical scientist reporting the results of urine and tumour MSI analyses.

### MMR immunohistochemistry

MMR IHC was performed in the histopathology department at the Newcastle upon Tyne Hospitals NHS Foundation Trust. Sections were stained with rabbit polyclonal antibodies against VENTANA® anti-MLH1 (M1) Mouse Monoclonal Primary Antibody (Roche Cat# 760-5091, RRID:AB_3669002), VENTANA anti-MSH2 (G219-1129) Mouse Monoclonal Primary Antibody (Roche Cat# 790-5093, RRID:AB_2936886), Monoclonal Rabbit Anti-Human PMS2, Clone EP51 (Agilent Cat# M3647, RRID:AB_3331634) and MSH6 antibody [EPR3945] (Abcam Cat# ab92471, RRID:AB_2144959). Analyses were performed by an NHS consultant histopathologist, to whom the results of MSI analyses were available. Tumours were defined as MMRd if there was loss of expression of one or more of MLH1, MSH2, MSH6 or PMS2. Tumours with expression of MLH1, MSH2, MSH6 and PMS2 were classified as MMR proficient (MMRp).

### Reference standard tests

Cohort 1: As the MSI-Plus assay was developed for CRC testing, we used MMR IHC as the reference standard for the performance of tumour MSI analysis for patients with symptomatic UTUC. The reference standard for pre-operative urine MSI analysis was matched tumour MSI status. The sequence of investigations (pre-operative urine MSI analysis followed by tumour MSI analysis, followed by tumour MMR IHC) meant that the results of the reference standard were not available at the time of reporting the results of the index test under comparison.

Cohort 2: For asymptomatic individuals with a diagnosis of *MSH2*-Lynch syndrome, the reference for MSI-H urine results was histological confirmation of urothelial carcinoma following tissue resection or biopsy. The reference for MSS urine results used medical records ± patient correspondence to confirm whether a cancer diagnosis had been made after at least 12 months follow up from urine sampling.

### Ethics

Ethical approval was granted as a regional service development project by Newcastle & North Tyneside Research Ethics Committee (Study: MRendometrial cancer/98/3/24). Written informed consent was obtained from all participants.

### Statistics

Sample size was determined by the patient population of The Newcastle Hospitals NHS Foundation Trust and North East and North Cumbria regional Genetics Service for cohorts 1 and 2 respectively, with the study design based upon estimations of the frequency of MMRd urothelial carcinoma diagnoses within these patient populations, calculated using a Clopper-Pearson binomial distribution.

Cohort 1 eligibility required diagnosis of UTUC following histological examination of a nephroureterectomy specimen. Over the study recruitment period (1/12/22 to 1/10/24), approximately 50 cases of UTUC were anticipated following local referral statistics, providing >80% probability for the cohort to include at least three MMRd tumours and >60% probability of at least four MMRd tumours, based on a previously reported frequency of 8.4%.^7^

Cohort 2 eligibility required a genetic diagnosis of Lynch syndrome associated with a constitutional (germline) likely pathogenic/pathogenic *MSH2* variant, an age of 30–75 years, and no cancer diagnosis in the past 12 months.^16^ From the 142 individuals with a diagnosis of *MSH2*-Lynch syndrome eligible for the study within our region, it was anticipated that approximately 70 individuals would participate (∼50% uptake, representing the highly engaged population in our region). Within a cohort of this size, there was >80% probability of at least one urothelial carcinoma diagnosis and >60% probability of two urothelial carcinoma diagnoses based on a 0.75% annual incidence rate of urothelial carcinoma among individuals with a diagnosis of *MSH2*-Lynch syndrome aged 40–75 years calculated from PLSD data and the assumption that the lead time for early detection of asymptomatic urothelial carcinoma can be 4 years or more ahead of conventional diagnosis.^5^^,^^17^

The methodology did not include randomisation or a formal blinding procedure, but the results of the reference standard were not available at the time of reporting pre-operative urine MSI analyses for cohort 1, or primary urine sample analyses for cohort 2.

Analyses used R version 4.4.2 (https://www.r-project.org/), with figures produced using ggplot2. Version 3.5.1^18^ Evaluation of the MSI assay used sensitivity, specificity, negative predictive value and positive predictive value (NPV/PPV) with 95% confidence intervals also calculated using a Clopper-Pearson binomial distribution. Receiver Operating Characteristic (ROC) plots and Area Under the Curve (AUC) values were generated for cohorts 1 and 2, with calculations for cohort 2 based upon the primary urine sample MSI scores, using the pROC R package. Version 1.18.0.^19^

### Role of funders

The study sponsors were not involved in the study design, data collection, data analyses or in the writing of this report.

## Results

A total of 54 consecutive patients with UTUC consented to be part of the project, but four were excluded with no evidence of UTUC on histology (cohort 1, Fig. 1, Table 1). Of 142 asymptomatic individuals with *MSH2*-Lynch syndrome contacted, 81 (57%) agreed to participate in the study (cohort 2, Fig. 1, Table 1) with one individual subsequently excluded (case A, see below).

**Table 1:** Participant demographics.

Participant demographics	Unselected patients with a diagnosis of UTUC (n = 50)	Asymptomatic individuals with a diagnosis of *MSH2-*LS (n = 81)
Age		
Median (range)	74 years (32–87 years)	57 years (32–75 years)
30–44 years	1	16
45–59 years	9	30
60–74 years	15	33
≥75 years	25	2
Sex		
Male	30	36
Female	20	45
Ethnicity		
White	41	80
Non-White	0	1
Not known	9	0
Previous hysterectomy		
Yes	n/a	41
No	n/a	4

Demographic details are provided for cohort 1 (50 unselected cases of UTUC undergoing nephroureterectomy) and cohort 2 (81 individuals with a diagnosis of MSH2-LS). Abbreviations used: **LS**, Lynch syndrome; **UTUC**, upper tract urothelial carcinoma.

### Cohort 1: unselected UTUC

Pre-operative urines and matched tumour DNA samples from 50 consecutive patients with UTUC were analysed by the MSI-Plus assay (tumour characteristics are provided in Supplementary Table S1). Tumour MMR IHC was performed for 49/50 UTUC tumour samples, as there was insufficient residual tumour tissue available for MMR IHC in one case. None of the participants were known to have Lynch syndrome at presentation, though case 1.1 had synchronous MMRd CRC (resected at the same time as nephroureterectomy) and a pending genetics referral.

**Fig. 2:**
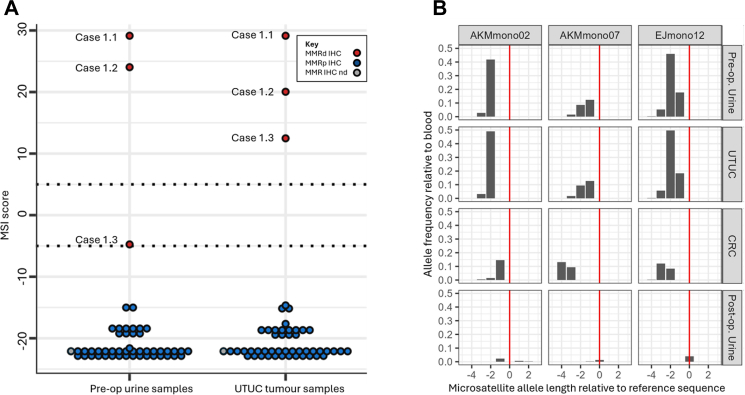
**MSI analysis of paired urine and tumour samples and tumour MMR IHC analyses for 50 unselected patients with UTUC undergoing nephroureterectomy (A) and normalised microsatellite allele length and allele frequencies for case 1.1 (B).** (A) MSI scores are shown for pre-operative urines (left) and matched tumours (right) from 50 patients with UTUC (Upper Tract Urothelial Carcinoma—cohort 1). MSI scores >0 are classified as MSI-H, scores <0 are classified as MSS. The dotted lines at −5 and +5 highlight the borderline range for repeat testing used by the clinical laboratory. For urine MSI analysis, borderline scores were considered MSI-H if a repeat was not available or the repeat result remained in the borderline range. KEY Red symbol: **MMRd** IHC—mismatch repair deficiency using ImmunoHistoChemistry; blue symbol: **MMRp** IHC—mismatch repair proficiency using ImmunoHistoChemistry; grey symbol (one case): **MMR** IHC **nd**—MisMatch Repair testing using ImmunoHistoChemistry **not done**. (B) Microsatellite alleles are presented along the x-axis as a length in nucleotides relative to the reference sequence: The ‘0’ allele indicates the microsatellite has a length equal to the reference sequence, whilst the negative alleles and the positive alleles represent deletions and insertions of the given number of nucleotides, respectively. The ‘0’ allele is marked by a vertical red line across plots. The y-axis represents the allele frequency as a proportion of sequencing reads. Given microsatellites are prone to amplification and sequencing error, each allele frequency was normalised by subtracting the respective allele frequency from the case 1.1 blood cfDNA sample (assuming blood cfDNA represents non-neoplastic tissue and so has allele distributions representing background error). Each column of plots represents an individual MSI marker, with three of the fourteen markers selected for this figure (AKMmono02, AKMmono07 and EJmono12, all markers shown in Supplementary Fig. S1). Each row represents one sample, which can be identified by the label to the right.

The MSI-Plus assay classified three UTUC tumour samples as MSI-H (6%; cases 1.1–1.3) and 47 as MSS (94%). The three MSI-H tumours were MMRd by IHC (100% sensitivity, CIs 29.2–100.0%), and the 46 MSS tumours with sufficient tissue available for IHC were MMRp (100% specificity, CIs 92.3–100.0%); Supplementary Table S2). Urine MSI-Plus analysis found the three patients with an MSI-H tumour to have MSI-H/borderline urines, whilst the 47 patients with an MSS tumour all had MSS urines (Fig. 2, Supplementary Table S2).

MMR IHC of the UTUC in case 1.1 showed loss of MSH2 and MSH6 expression, whilst loss of MSH6 was detected in cases 1.2 and 1.3. Variant allele frequencies (VAFs) for the 14 MSI markers correlated between urine and matched UTUC for cases 1.1–1.3, demonstrating the urine MSI signal originated from the UTUC (and not the CRC in case 1.1, Fig. 2, Supplementary Figures S1-3) and post-operative urine analyses were MSS (Supplementary Table S2). Constitutional genetic testing confirmed Lynch syndrome in each case, with pathogenic variants detected in *MSH2* (case 1.1) and *MSH6* (cases 1.2 and 1.3).^16^

Among symptomatic patients with a diagnosis of UTUC, urine MSI analysis detected MSI-H UTUC with 100% sensitivity (95% CI 29.2–100.0%), 100% specificity (95% CI 92.5–100.0%), 100% PPV (95% CI 29.2–100.0%), and 100% NPV 100% (95% CI 92.5–100.0%), giving a ROC AUC of 1.000 (Supplementary Figure S4). Sex disaggregated results are provided in Supplementary Table S3.

### Cohort 2: asymptomatic *MSH2*-Lynch syndrome

**Fig. 3:**
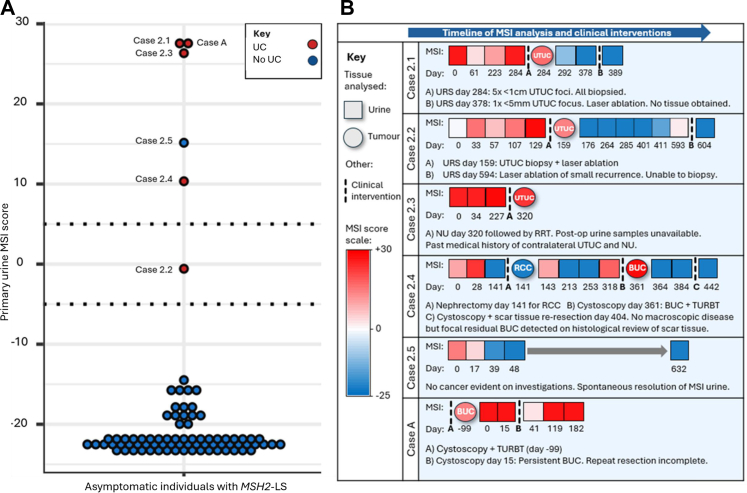
**Initial urine MSI analysis results from 81 individuals with a diagnosis of *MSH2*-Lynch syndrome (A) and a timeline of urine and tumour MSI analysis results for participants with an MSI-High urine result (B).** (A) The y axis represents the output score of the Newcastle MSI-Plus assay. Scores >0 are classified as MSI-H, scores <0 are classified as MSS. The dotted lines at −5 and +5 highlight the borderline range for repeat testing used by the clinical laboratory. For urine MSI analysis, borderline scores were considered MSI-H if a repeat was not available or the repeat result remained in the borderline range. Each of the 81 dots represents the initial urine MSI score for each of the 81 individuals with a diagnosis of *MSH2*-Lynch syndrome. (B) The six individuals with a diagnosis of *MSH2*-Lynch syndrome whose primary urine sample MSI analysis gave an MSI-H result are shown as individual rows. Each coloured square represents a separate urine MSI analysis, with the day the sample was taken provided directly underneath. Tumour sample analyses are shown as circles. The date of first urine MSI analysis is day ‘0’ in all cases. The colour of each square/circle represents the output MSI score according to the red (MSI-H) to blue (MSS) scale shown in the key. The timing of clinical interventions including treatment/resection of tumours is shown as dashed lines. Abbreviations used: **BUC**, bladder urothelial carcinoma; **MSI**, microsatellite instability; **MSI-H**, high levels of microsatellite instability; **MSS**, microsatellite stable; **NU**, nephroureterectomy; **RCC**, renal cell carcinoma; **RRT**, renal replacement therapy; **TURBT**, transurethral resection of bladder tumour; **URS**, ureterorenoscopy; **UTUC**, upper tract urothelial carcinoma.

Urine MSI analysis of the 81 asymptomatic individuals with a diagnosis of *MSH2*-Lynch syndrome identified six cases with an MSI-H result (Fig. 3). One who had developed haematuria, was diagnosed with a bladder urothelial carcinoma (BUC) and was treated by transurethral resection of bladder tumour (TURBT) between agreeing to participate in the study and their scheduled appointment. This individual is therefore distinguished from the main cohort as case A. Of the remaining five cases with an MSI-H urine result, four were found to have MSI-H urothelial carcinoma (cases 2.1–2.4), with histological confirmation a median 281 (range 159–361) days after initial urine MSI analysis. One case with an MSI-H urine result showed no evidence of cancer (case 2.5). Analysis of MSI marker VAFs confirmed the urothelial carcinoma to be the source of the urine MSI signal in each case of urothelial carcinoma (Supplementary Figures S5-10). Plasma cfDNA MSI analysis was MSS for cases 2.1 to 2.5, and was not performed for case A. Gynaecological referral was not indicated for the female MSI-H cases due to previous prophylactic hysterectomy. Details of each case with an MSI-H urine result are summarised below with a timeline of MSI analyses (Fig. 3B, see also Supplementary Table S4). After a minimum follow-up period of 12 months, none of the participants with an initial MSS urine had a diagnosis of urothelial carcinoma or endometrial cancer, nor reported any symptoms at last contact (median time from first sample collection to last follow up 567 days, range 465–764 days). 70/75 provided a follow up postal urine sample, all of which were MSS (median 563 days from first sample collection, range 468–764 days). MSI scores from all analyses are provided in Supplementary Table S5 and Supplementary Figure S11.

For case 2.1 and 2.2, early detection of low risk UTUC allowed treatment via URS. In case 2.1, URS identified multifocal disease with five tumours of <1 cm diameter in the renal pelvis and each was biopsied with a diagnosis of low grade pTa UTUC. All urines following the biopsies were MSS. A second URS revealed a single <5 mm focus of disease, which underwent ablation. In case 2.2, tumour resection and laser ablation were performed at the first URS and the tumour histology revealed a low grade pTa UTUC. Post-resection urine MSI analyses were MSS until day 593, when urine MSI analysis was MSI-H immediately prior to the detection of a small recurrence on follow up URS. The MSI-H signal again resolved following treatment.

In case 2.3, the patient had a contralateral UTUC resected 10 years previously. Following their MSI-H urine result, a UTUC was suspected based on CT appearance and cytology of washings taken at URS, and histopathology subsequently confirmed pT3 high grade UTUC. The patient underwent a second nephroureterectomy, followed by renal replacement therapy; further urine testing was, therefore, not possible.

In case 2.4, a renal cell carcinoma (RCC) was diagnosed following identification of a renal mass on ultrasound. The RCC was removed via nephrectomy but found to be MSS in all tumour blocks. Post-operative urines had variable MSI results and a cystoscopy detected a high grade pT1 MSI-H BUC, which was treated by TURBT. Subsequent urines were MSS.

The MSI-H signal of the first urine in case 2.5 reduced and became MSS over 6 weeks of urine sampling. The only abnormality on urological investigations was a complex (Bosniak 2F) renal cyst with an unchanged appearance from imaging performed 4 years previously. In the 18 months following urine testing, the patient developed an MSS breast ductal carcinoma in situ but did not report any urological symptoms and had an MSS urine sample 21 months after the first urine.

The patient in case A was unaware of ongoing disease at the time of postal urine sampling three months after TURBT. Cystoscopy confirmed residual/recurrent pT1 BUC, consistent with their MSI-H urine. Complete TURBT has not been possible due to disease location and a decision was made against radical cystectomy based on patient co-morbidities. Repeat urines remain MSI-H.

Among asymptomatic individuals with a diagnosis of *MSH2*-Lynch syndrome (excluding case A), urine MSI analysis detected asymptomatic urothelial carcinoma with 100% sensitivity (95% CI 39.8–100.0%), 98.7% specificity (95% CI 92.9–99.9%), 80% PPV (95% CI 28.4%-99.5%), and 100% NPV (95% CI 95.2–100.0%) (Supplementary Table S6). The ROC AUC value based upon primary urine sample analyses was 0.995 (Supplementary Figure S12). Sex disaggregated results are provided in Supplementary Table S3.

### Comparison with existing methods

Urinalysis or urine microscopy results were available for 55/80 individuals with *MSH2*-Lynch syndrome. Microhaematuria was detected in 25% of cases found to have urothelial carcinoma (1/4, case 2.3) and 6% (3/51) of cases where a cancer was not diagnosed (Table 2). The urine microscopy RBCC was 0/mm^3^ prior to URS for cases 2.1 and 2.2, and urinalysis performed in clinic did not detect haematuria for case 2.4.

**Table 2:** Comparison of urine MSI analysis results and the detection of microhaematuria in cohort 2.

	Urine MSI analysis		Microscopic haematuria	
	MSI-H	MSS	Present	Absent
UC (n = 4)	4	0	1	3
No cancer (n = 51)	1	50	3	48

Numbers consist of the 52 patients for whom urinalysis was performed in clinic at the time of urine MSI analysis (which includes two of the individuals found to have an MSI-High urine result, cases 2.3 and 2.5), as well as the three individuals found to have a urothelial carcinoma following postal urine MSI analysis who had urine microscopy performed as part of their pre-operative assessment at a later stage of their clinical work up (cases 2.1, 2.2 and 2.4). Abbreviations used: **MSI**, microsatellite instability; **MSI-H**, high levels of microsatellite instability; **MSS**, microsatellite stable; **UC**, urothelial carcinoma.

Abdominal ultrasound was performed for 44/52 individuals with *MSH2*-Lynch syndrome seen in clinic. Of these, the only case found to have cancer was case 2.4, for whom the ultrasound identified the RCC but not the BUC. Ultrasound detected benign abnormalities in nine cases (Supplementary Table S7).

## Discussion

UcfDNA MSI analysis to screen for MMRd urothelial carcinoma gave encouraging results in both cohorts, with the detection of asymptomatic urothelial carcinoma in four individuals with a diagnosis of *MSH2*-Lynch syndrome, highlighting the potential benefit for this high-risk group.

Amongst cohort 1 patients with UTUC, urine MSI analysis detected all three MSI-H tumours, achieving 100% diagnostic accuracy. Whilst a larger cohort is needed to refine accuracy statistics, our results suggest urine MSI analysis could be used as a minimally invasive liquid biopsy test for the presence of a microsatellite unstable cancer. This would provide urology/oncology teams with UTUC MMR status prior to treatment, enable prompt screening for Lynch syndrome, and provide the cancer multi-disciplinary team with an early indication of potential response to adjuvant immune checkpoint inhibition, for which there is increasing evidence in MMRd UC.^20^^,^^21^

Current UK practice includes MMR tumour testing of all CRC and endometrial cancer cases to screen for Lynch syndrome, but not UTUCs despite the known association with Lynch syndrome.^11^^,^^22^^,^^23^ Here, we diagnosed three new cases of Lynch syndrome, two of whom had MSH6 pathogenic variants. Since MSH6-Lynch syndrome is less likely to present with CRC, screening of UTUC diagnoses may be particularly valuable for this Lynch syndrome subtype.^24^ The 6% frequency of MSI and Lynch syndrome in our UTUC cohort is consistent with the 8.4% and 6.5% frequencies reported in a recent review collating results from 2427 and 783 individuals with UTUC undergoing MSI and Lynch syndrome testing respectively.^7^

The Newcastle MSI-Plus assay was primarily developed for the analysis of CRC tumour samples and is clinically validated for this purpose.^12^ There are, however, reasons to predict that the assay can be used on a tumour/tissue agnostic basis. In particular, the MSI markers analysed can accurately detect the low-level MSI in the blood of constitutional mismatch repair deficiency syndrome,^25^ and have proven to be sensitive and specific across several non-colorectal tumour types, including urothelial, endometrial, and sebaceous tumours.^26^, ^27^, ^28^ Here, tumour MSI testing and MMR IHC analyses demonstrated 100% concordance in the 49 cases of UTUC where both techniques were performed. These data, along with ongoing analyses, will inform the continual development of the assay for urine and urothelial carcinoma tumour analyses. In particular, case 1.3 highlighted that MSI scores within the ‘borderline’ range of −5 to +5 can occur in urine samples from patients with an MMRd UC, and the MSI classifier may, therefore, benefit from re-training or adjustment of classification thresholds for urothelial carcinoma surveillance.

In the *MSH2*-Lynch syndrome cohort, we detected urothelial carcinoma in four asymptomatic individuals with 100% sensitivity and 98.7% specificity. Early-stage (Ta/T1) disease was detected in three cases, and for two patients the early detection of UTUC allowed endoscopic treatment. In case 2.3, the patient now requires renal replacement therapy following bilateral nephroureterectomies for UTUC. This highlights the morbidity that can be associated with UTUC in Lynch syndrome, and the potential benefit of kidney-sparing therapy following early diagnosis. Our initial estimates of sensitivity and specificity suggest urine MSI analysis is a more accurate screening test for Lynch syndrome urothelial carcinoma than urinalysis or urine cytology: Urinalysis was shown to have 0% sensitivity and 0% specificity, and urine cytology has been shown to have only 29% sensitivity.^9^^,^^10^ Amongst the four patients with urothelial carcinoma diagnoses in this study, microhaematuria was detected in one (case 2.3), and voided-urine cytology was negative in the two cases where it was performed (cases 2.2 and 2.4). Case A and case 2.2 also demonstrated how urine MSI analysis can detect residual/recurrent disease, and further work could explore urine MSI analysis for post-treatment monitoring.

There was one false positive result (case 2.5), with a transient MSI-H signal detected in the patient's urine samples. The cause was unclear and data from a larger cohort of individuals with Lynch syndrome will be required to accurately determine the false positive rate of urine MSI analysis in Lynch syndrome. Potential causes of false positive results could include technical error (considered unlikely in case 2.5 based on the repeat MSI-H urine sample on day 17), the presence of non-malignant MMRd foci in the urogenital tissues, or the spontaneous regression of an MMRd urogenital cancer following immune recognition as has been reported in MMRd CRC.^29^^,^^30^ Analysis of ucfDNA from a control population of individuals without a diagnosis of cancer or Lynch syndrome would be of interest to better define baseline signals and understand the potential causes of false positives.

Whilst there were no false negative results from initial urine MSI analysis, MSS urine samples were found in the presence of an MSI-H urothelial carcinoma during follow up testing prior to tumour resection/ablation. In case 2.1 the MSI-H signal in the urine disappeared following the patient's initial URS, when biopsies were taken from the 5 small foci of disease and repeat urine MSI analysis was MSS when a second URS identified one ∼5 mm focus of disease. We presume the biopsies removed the bulk of the disease leaving insufficient tumour to detect an MSI-H urine signal. In case 2.4, the patient's urine MSI results were fluctuant, with 3/7 samples giving an MSS result prior to the removal of the MSI-H BUC. We speculate that cfDNA shed from the MSS RCC or during surgical wound healing may have influenced the MSS results,^31^ but ultimately the reason for the variable results is unclear. Serial sampling of additional MMRd urothelial carcinoma patients is needed to understand the biological, sampling, and technical variables that can impact assay sensitivity.

Notably, the incidence of urothelial carcinoma in our *MSH2*-Lynch syndrome cohort (excluding case A) was 5% (4/80, 95% CI 1.4–12.3%). This is higher than expected based on the PLSD, where the highest annual incidence ratio for any age, gender or genotype bracket was 0.0259 (around 2.6%, females with a diagnosis of *MSH2*-Lynch syndrome aged 75–79 years).^11^ We suspect this reflects our unscreened population, as the latent period of urothelial carcinoma is not well established. Indeed, case 2.4 demonstrated that urine MSI testing may detect a signal from a BUC for at least 12 months without the tumour becoming symptomatic. It is also possible that our small sample population contained a higher burden of disease by chance.

A strength of this study is the use of two complimentary well-defined, inclusive patient populations, with cohort 2 directly representing the Lynch syndrome population most likely to be offered regular urothelial carcinoma screening in the future. In addition, testing took place within NHS laboratories, with our results therefore reflecting how the Newcastle MSI-Plus assay would perform if adopted for routine urothelial carcinoma surveillance of individuals with a diagnosis of *MSH2*-Lynch syndrome or for MMRd testing of unselected UTUC.

A key limitation is the modest sample size of 81 individuals with *MSH2*-Lynch syndrome recruited from a single institution, which is reflected in the wide confidence intervals for assay sensitivity and PPV. A larger, multi-centre study is needed to refine these accuracy statistics. However, our results are very promising despite this, and it is noteworthy that urine MSI analysis already provides significantly greater sensitivity and PPV than the alternative, clinically-available screening methods of haematuria and urine cytology.^9^^,^^10^ Therefore, this pilot study represents an important advance, on which future research and diagnostic practice can be built. Another limitation is the initial focus on *MSH2*-Lynch syndrome, which was pursued to maximise urothelial carcinoma diagnoses and as the population most in need of intervention; data are also needed for the other Lynch syndrome genotypes. To this end, we have now approached the approximately 250 individuals with known diagnoses of *MLH1*- and *MSH6*-Lynch syndrome in our regional population of 3 million and will extend the offer to the around 1500 Lynch syndrome carriers being followed by our team as part of the CaPP3 aspirin dose non-inferiority trial [EUdract no. 2014-000411-14].

Importantly, this work does not address the potential for ucfDNA MSI analysis to detect MMRd endometrial cancer, as demonstrated previously.^4^ Typically, Lynch syndrome endometrial cancer surveillance is not currently offered due to a lack of evidence that methods such as ultrasound, biopsy and/or hysteroscopy result in improved outcomes.^8^^,^^32^ Our Lynch syndrome cohort only contained four individuals with an endometrium, since most had undergone risk-reducing hysterectomy. This is a limitation; first because our results cannot shed light on the ability to detect endometrial cancer, and second because non-neoplastic MMRd endometrial cancer glands have been reported in individuals with Lynch syndrome without a cancer diagnosis,^33^ which could increase false positive rates, with one study identifying MMRd foci in 47% of Lynch syndrome endometrial cancer mucosa samples.^29^ This work also does not determine the range of malignancies detectable using urine MSI analysis, but we expect that our methodology would only detect urogenital MMRd cancers. This is supported by case 1.1 where there was no evidence of the synchronous CRC MSI signal in the urine based on analysis of the variant alleles.

There is widespread interest in liquid biopsy-based cancer screening, with amplicon sequencing and target capture/deep-sequencing approaches as well as the use of microsatellite markers to detect copy number variation in ucfDNA all having been previously explored for urothelial carcinoma detection.^34^^,^^35^ Our approach exploits the high frequency of MMRd amongst Lynch syndrome cancers to use MSI analysis as the primary method of urothelial carcinoma detection.^36^ The MSI-Plus assay is low cost and high-throughput, fully automatable, and can analyse <1 ng of sample DNA,^4^^,^^14^ making it particularly suited for routine and repeat surveillance using ucfDNA. Use of MSI alone may limit sensitivity since up to 15% of urothelial carcinoma occurring in Lynch syndrome carriers may be MMRp.^36^ Assay development is therefore ongoing to include urothelial carcinoma mutation hotspots with a view to broadening the relevance of the assay as a diagnostic tool, whilst minimising impact upon cost.

In summary, we have demonstrated the feasibility of repurposing the Newcastle MSI-Plus assay for the detection of asymptomatic MMRd urothelial carcinoma using ucfDNA. Our data suggest this approach to Lynch syndrome urothelial carcinoma screening could have high levels of sensitivity and specificity and work is therefore underway to develop a larger dataset to further define the accuracy of the assay in the high-risk Lynch syndrome patient population. In addition, we have demonstrated how the same non-invasive approach could be used to screen individuals with UTUC for Lynch syndrome.

## Contributors

All authors read and approved the final version of this manuscript. Rebecca Hall and Richard Gallon both accessed and reviewed all of the underlying data. The authors each made the following contributions to this work. Rebecca Hall–conceptualisation, data curation, funding acquisition, investigation, formal analysis, methodology, project administration (clinical), visualisation, writing the original draft. Richard Gallon—conceptualisation, data curation, formal analysis, investigation, methodology, software, visualisation, writing-review and editing. Christine Hayes—conceptualisation, methodology, investigation, project administration, writing. Patricia Herrero-Belmonte—investigation, data curation, writing—review and editing. Rachel Phelps—conceptualisation, investigation, writing—review and editing. Donna Job—conceptualisation, project administration. Ruth Wake–conceptualisation, project administration. Mary Ferrier—investigation, resources, writing—review and editing. Helen Turner—investigation, resources, writing—review and editing. Rachel O'Donnell—resources, writing—review and editing. Arjun Nambiar—supervision, writing—review and editing. Bhavan Rai—conceptualisation, methodology, supervision, writing—review and editing. Richard Martin—conceptualisation, supervision, writing—review and editing. Ciaron McAnulty—resources, supervision, writing—review and editing. Mauro Santibanez-Koref—conceptualisation, software, methodology, supervision, writing—review and editing. Rakesh Heer—conceptualisation, methodology, supervision, writing—review and editing. Michael Jackson—conceptualisation, methodology, supervision, writing—review and editing. John Burn—conceptualisation, funding acquisition, methodology, project administration, supervision, writing—review and editing.

## Data sharing statement

MSI-Plus assay FASTQ files are freely available from the European Nucleotide Archive using study identifier PRJEB85932 (https://www.ebi.ac.uk/ena/browser/view/PRJEB85932).

## Declaration of interests

R. Gallon, J. Burn, M. S. Jackson, and M. Santibanez-Koref are named inventors on patents covering the microsatellite instability markers analysed: WO/2018/037231 (published March 1, 2018), WO/2021/019197 (published February 4, 2021), and GB2114136.1 (filed October 1, 2021). J. Burn is chair and has a minor shareholding in QuantuMDx ltd: the company funded the first PhD studentship regarding the development of the Newcastle MSI-Plus assay and was granted a profit share of any income beyond a set threshold by the university in return for relinquishing rights to develop the assay commercially. J Burn and R Gallon were named as co-applicants on the NHS Small Business Research Initiative award NU-008478 SBRIC01P3040 used by Newcastle Hospitals to develop the MSI-Plus assay for diagnostic tumour testing. R Gallon received support for attending meetings and/or travel PCR Biosystems Financial support to attend the European Congress of Pathology 7th to 11th September 2024. The other authors declare no conflicts of interest.
