# Supplementary material for: Detection of urothelial carcinoma in Lynch syndrome using microsatellite instability analysis of urine cell-free DNA
Source: eBioMedicine. 2025 Oct 25;121:105969. doi: 10.1016/j.ebiom.2025.105969 (PMC12595274; doi:10.1016/j.ebiom.2025.105969)
Supplement: Supplementary Tables [file mmc1.docx]

**Supplementary Table 1: Upper tract urothelial carcinoma (UTUC) cohort tumour characteristics**

| **UTUC cohort: Tumour characteristics (n=50)** | |
| --- | --- |
| **Stage:** pTa pT1 pT2 pT3 | **Number** 20 9 7 14 |
| **Grade (WHO 2022):** Low grade Low grade with focal high grade High grade | 12 6 32 |
| **Histological subtype:** Pure urothelial Squamous divergence Sarcomatoid urothelial | 47 1 2 |
| **Site:** Ureter Renal pelvis or calyces Multifocal | 23 21 6 |

**Supplementary Table 1: Upper tract urothelial carcinoma (UTUC) cohort tumour characteristics**

Tumour staging is provided according to the Tumour Node Metastasis (TNM) staging classification. Tumour grade follows the histological classification of UCs published in 2022.

Abbreviations: UTUC Upper tract urothelial carcinoma, WHO World Health Organization

**Supplementary Table 2 (1/3): Matched MSI scores and MMR IHC for unselected cases of UTUC**

| **Case ID** | **Gender** | **Sample type** | **MSI result** | **MSI score** | **MMR IHC result** |
| --- | --- | --- | --- | --- | --- |
| Case1.1 | Female | Pre-op urine | MSI-H | 29.143 | n/a |
|  |  | Tumour (CRC) | MSI-H | 27.227 | Not performed |
|  |  | Tumour (UTUC) | MSI-H | 29.143 | Loss of MSH2 and MSH6 |
|  |  | Post-op urine | MSS | -22.38 | n/a |
| Case1.2 | Male | Pre-op urine | MSI-H | 24.036 | n/a |
|  |  | Tumour | MSI-H | 20.045 | Loss of MSH6 |
|  |  | Post-op urine | MSS | -22.38 | n/a |
| Case1.3 | Male | Pre-op urine | Borderline/MSI-H | -4.753 | n/a |
|  |  | Tumour | MSI-H | 12.481 | Loss of MSH6 |
|  |  | Post-op urine | MSS | -22.247 | n/a |
| Case1.4 | Female | Pre-op urine | MSS | -22.598 | n/a |
|  |  | Tumour | MSS | -22.996 | Proficient MMR |
| Case1.5 | Male | Pre-op urine | MSS | -22.38 | n/a |
|  |  | Tumour | MSS | -22.38 | Proficient MMR |
| Case1.6 | Male | Pre-op urine | MSS | -18.318 | n/a |
|  |  | Tumour | MSS | -18.638 | Proficient MMR |
| Case1.7 | Female | Pre-op urine | MSS | -19.005 | n/a |
|  |  | Tumour | MSS | -18.964 | Proficient MMR |
| Case1.8 | Female | Pre-op urine | MSS | -21.623 | n/a |
|  |  | Tumour | MSS | -17.66 | Proficient MMR |
| Case1.9 | Male | Pre-op urine | MSS | -22.38 | n/a |
|  |  | Tumour | MSS | -15.411 | Proficient MMR |
| Case1.10 | Male | Pre-op urine | MSS | -18.163 | n/a |
|  |  | Tumour | MSS | -21.849 | Proficient MMR |
| Case1.11 | Male | Pre-op urine | MSS | -18.695 | n/a |
|  |  | Tumour | MSS | -18.695 | Proficient MMR |
| Case1.12 | Female | Pre-op urine | MSS | -19.443 | n/a |
|  |  | Tumour | MSS | -19.443 | Proficient MMR |
| Case1.13 | Male | Pre-op urine | MSS | -21.849 | n/a |
|  |  | Tumour | MSS | -21.849 | Proficient MMR |
| Case1.14 | Male | Pre-op urine | MSS | -21.849 | n/a |
|  |  | Tumour | MSS | -22.333 | Proficient MMR |
| Case1.15 | Male | Pre-op urine | MSS | -22.616 | n/a |
|  |  | Tumour | MSS | -22.6 | Proficient MMR |
| Case1.16 | Male | Pre-op urine | MSS | -22.247 | n/a |
|  |  | Tumour | MSS | -22.598 | Proficient MMR |
| Case1.17 | Male | Pre-op urine | MSS | -21.849 | n/a |
|  |  | Tumour | MSS | -22.247 | Proficient MMR |
| Case1.18 | Male | Pre-op urine | MSS | -22.127 | n/a |
|  |  | Tumour | MSS | -22.327 | Proficient MMR |
| Case1.19 | Female | Pre-op urine | MSS | -15.234 | n/a |
|  |  | Tumour | MSS | -22.324 | Proficient MMR |
| Case1.20 | Female | Pre-op urine | MSS | -22.334 | n/a |
|  |  | Tumour | MSS | -22.619 | Proficient MMR |

**Supplementary Table 2 (2/3): Matched MSI scores and MMR IHC for unselected cases of UTUC**

| **Case ID** | **Sex** | **Sample type** | **MSI result** | **MSI score** | **IHC MMR result** |
| --- | --- | --- | --- | --- | --- |
| Case1.21 | Male | Pre-op urine | MSS | -22.334 | n/a |
|  |  | Tumour | MSS | -22.334 | Proficient MMR |
| Case1.22 | Female | Pre-op urine | MSS | -22.598 | n/a |
|  |  | Tumour | MSS | -19.648 | Proficient MMR |
| Case1.23 | Male | Pre-op urine | MSS | -23.129 | n/a |
|  |  | Tumour | MSS | -18.665 | Proficient MMR |
| Case1.24 | Male | Pre-op urine | MSS | -19.443 | n/a |
|  |  | Tumour | MSS | -14.658 | Proficient MMR |
| Case1.25 | Male | Pre-op urine | MSS | -18.389 | n/a |
|  |  | Tumour | MSS | -18.389 | Proficient MMR |
| Case1.26 | Male | Pre-op urine | MSS | -22.354 | n/a |
|  |  | Tumour | MSS | -22.086 | Proficient MMR |
| Case1.27 | Male | Pre-op urine | MSS | -22.598 | n/a |
|  |  | Tumour | MSS | -22.598 | Proficient MMR |
| Case1.28 | Female | Pre-op urine | MSS | -23.129 | n/a |
|  |  | Tumour | MSS | -23.129 | Proficient MMR |
| Case1.29 | Female | Pre-op urine | MSS | -22.127 | n/a |
|  |  | Tumour | MSS | -22.32 | Proficient MMR |
| Case1.30 | Female | Pre-op urine | MSS | -21.849 | n/a |
|  |  | Tumour | MSS | -21.849 | Proficient MMR |
| Case1.31 | Male | Pre-op urine | MSS | -22.778 | n/a |
|  |  | Tumour | MSS | -22.342 | Proficient MMR |
| Case1.32 | Male | Pre-op urine | MSS | -21.849 | n/a |
|  |  | Tumour | MSS | -21.849 | Proficient MMR |
| Case1.33 | Male | Pre-op urine | MSS | -22.598 | n/a |
|  |  | Tumour | MSS | -22.598 | Proficient MMR |
| Case1.34 | Male | Pre-op urine | MSS | -23.129 | n/a |
|  |  | Tumour | MSS | -19.443 | Proficient MMR |
| Case1.35 | Female | Pre-op urine | MSS | -19.222 | n/a |
|  |  | Tumour | MSS | -19.222 | Proficient MMR |
| Case1.36 | Male | Pre-op urine | MSS | -23.129 | n/a |
|  |  | Tumour | MSS | -23.129 | Proficient MMR |
| Case1.37 | Female | Pre-op urine | MSS | -22.056 | n/a |
|  |  | Tumour | MSS | -18.392 | Proficient MMR |
| Case1.38 | Female | Pre-op urine | MSS | -22.38 | n/a |
|  |  | Tumour | MSS | -22.38 | Proficient MMR |
| Case1.39 | Male | Pre-op urine | MSS | -22.622 | n/a |
|  |  | Tumour | MSS | -22.325 | Proficient MMR |
| Case1.40 | Female | Pre-op urine | MSS | -22.596 | n/a |
|  |  | Tumour | MSS | -22.312 | Insufficient tissue available |

**Supplementary Table 2 (3/3): Matched MSI scores and IHC MMR for unselected cases of UTUC**

| **Case ID** | **Sex** | **Sample type** | **MSI result** | **MSI score** | **IHC MMR result** |
| --- | --- | --- | --- | --- | --- |
| Case1.41 | Male | Pre-op urine | MSS | -22.38 | n/a |
|  |  | Tumour | MSS | -22.38 | Proficient MMR |
| Case1.42 | Female | Pre-op urine | MSS | -23.129 | n/a |
|  |  | Tumour | MSS | -23.129 | Proficient MMR |
| Case1.43 | Male | Pre-op urine | MSS | -23.129 | n/a |
|  |  | Tumour | MSS | -23.129 | Proficient MMR |
| Case1.44 | Female | Pre-op urine | MSS | -18.14 | n/a |
|  |  | Tumour | MSS | -14.92 | Proficient MMR |
| Case1.45 | Female | Pre-op urine | MSS | -22.317 | n/a |
|  |  | Tumour | MSS | -22.622 | Proficient MMR |
| Case1.46 | Male | Pre-op urine | MSS | -18.638 | n/a |
|  |  | Tumour | MSS | -18.648 | Proficient MMR |
| Case1.47 | Male | Pre-op urine | MSS | -23.129 | n/a |
|  |  | Tumour | MSS | -23.129 | Proficient MMR |
| Case1.48 | Female | Pre-op urine | MSS | -22.618 | n/a |
|  |  | Tumour | MSS | -22.316 | Proficient MMR |
| Case1.49 | Male | Pre-op urine | MSS | -14.788 | n/a |
|  |  | Tumour | MSS | -18.899 | Proficient MMR |
| Case1.50 | Female | Pre-op urine | MSS | -22.38 | n/a |
|  |  | Tumour | MSS | -22.38 | Proficient MMR |

**Supplementary Table 2: Matched MSI scores and IHC MMR for unselected cases of UTUC**

Cases are numbered from case 1.1 to case 1.50, with the three cases with mismatch repair deficient tumours being cases 1.1-1.3. MSI scores are provided for all urine and tumour analyses, with corresponding MMR IHC provided for all except case 1.40, for whom there was insufficient tumour tissue available for MMR IHC. Full details of the interpretation of MSI scores is provided in the main methodology: MSI scores within the borderline range of -5 to +5 were repeated where available, or considered MSI-High where a repeat was not possible (as in the case of pre-operative urine samples).

Abbreviations: MSI Microsatellite instability, MMR IHC Mismatch repair protein immunohistochemistry, UTUC upper tract urothelial carcinoma, CRC colorectal carcinoma, MSI-H high levels of microsatellite instability, MSS microsatellite stable

**Supplementary Table 3: Sex disaggregated results of MSI analysis for cohorts 1 and 2**

| **Unselected cases of UTUC undergoing NU (n=50)** | | | |
| --- | --- | --- | --- |
| **Tumour MSI testing using the Newcastle MSI-Plus assay** | | | |
|  | **Female** | **Male** | **Total(s)** |
| **Tumour MSI-High** | 1 | 2 | 3 |
| **Tumour MSS** | 19 | 28 | 47 |
| **Total(s)** | 20 | 30 | 50 |
| **Pre-operative urine MSI testing using the Newcastle MSI-Plus assay** | | | |
|  | **Female** | **Male** | **Total(s)** |
| **Urine MSI-High** | 1 | 2* | 3* |
| **Urine MSS** | 19 | 28 | 47 |
| **Total(s)** | 20 | 30 | 50 |
| **Asymptomatic individuals with a diagnosis of *MSH2*-LS (n=81)** | | | |
|  | **Female** | **Male** | **Total(s)** |
| **Urine MSI high** | 3 | 3** | 6 |
| **Urine MSS** | 42 | 33 | 75 |
| **Total(s)** | 45 | 36 | 81 |

**Supplementary Table 3: Sex disaggregated results of MSI analysis for cohorts 1 and 2**MSI scores >0 are classified as MSI-H, scores <0 are classified as MSS. The dotted lines at -5 and +5 highlight the borderline range for repeat testing used by the clinical laboratory. For urine MSI analysis, borderline scores were considered MSI-H if a repeat was not available or the repeat result remained in the borderline range. In the UTUC cohort, repeat pre-operative urine sample collection was not possible for case 1.3 and the borderline result was therefore treated as MSI-H as per our methodology: * therefore denotes the inclusion of this case where the initial urine MSI result fell within the borderline range of -5 to +5 in the MSI-H cohort. In the *MSH2*-LS cohort, **Denotes the inclusion of case A amongst cases with an MSI-High urine result. In this case a diagnosis of BUC had been made, and a TURBT performed, between agreeing to be part of the project and urine MSI analysis.

Abbreviations: MSI microsatellite instability, UTUC upper tract urothelial carcinoma, NU nephroureterectomy, LS Lynch syndrome, MSS microsatellite stable, TURBT Transurethral resection of bladder tumour

**Supplementary Table 4: Clinical investigation and tumour details of cases with an MSI-H urine result from the *MSH2*-LS cohort**

|  | **Age and sex** | **Cancer Y/N** | **Haematuria on urinalysis or urine microscopy** | **Urine cytology** | **Tumour site** | **Tumour stage** | **Histology** | **Cancer management** | **Other relevant information** |
| --- | --- | --- | --- | --- | --- | --- | --- | --- | --- |
| 2.1 | 60-74 years,  female | Y | No  (performed prior to URS) | Not performed | Renal pelvis (L) | TaN0M0 | Low grade (G2) papillary urothelial carcinoma (biopsy) | Endoscopic management to date. | Multifocal disease with 5 tumours of ~1cm diameter. |
| 2.2 | 45-59 years Male | Y | No  (performed prior to URS) | Negative  (wk 11) | Ureter (R) | TaN0M0 | Low-grade (G2) papillary urothelial carcinoma (biopsy) | Endoscopic management to date. |  |
| 2.3 | 60-74 years, male | Y | Yes (performed in pre-op assessment) | Negative  (wk 24)  Positive  (wk 39) | Renal pelvis (R) | T3N0M0 | High grade urothelial carcinoma | Nephroureterectomy followed by renal replacement therapy. | Previous UTUC and nephroureterectomy >10 years ago. |
| 2.4 | 60-74 years, female | Y | No (performed at time of urine MSI in clinic) | Not performed | 1. Renal parenchyma  2. Bladder | 1. T3aN0M0  2. TaN0M0 | 1. 1. Clear cell renal cell carcinoma 2. 2. High grade (G2/3) papillary urothelial carcinoma | 1. Nephrectomy  2. Transurethral resection of bladder tumour |  |
| 2.5 | ≥75 years, female | N | No (performed at time of urine MSI in clinic) | Negative | N/A | N/A | N/A | N/A | Renal cyst (Bosniak 2F) identified on renal ultrasound.  Diagnosis of DCIS of the breast 13 months following 1^st^ MSI-H urine result. DCIS tissue MSS. |
| A | ≥75 years, male | Y | N/A. Cancer diagnosed prior to project | N/A. Cancer diagnosed prior to project | Bladder | T1N0M0 | High grade (G3) papillary urothelial carcinoma | Transurethral resection of bladder tumour x 2 | Found to have persistent disease with involvement of left ureteric opening. |

**Supplementary Table 4: Clinical investigation and tumour details of cases with an MSI-H urine result from the *MSH2*-LS cohort**

Details are provided regarding the results of urinalysis for haematuria and urine cytology (where performed), tumour characteristics and clinical management undertaken for cases 2.1-2.5 and case A.

Abbreviations: MSI Microsatellite instability, LS Lynch syndrome, URS ureterorenoscopy, UTUC upper tract urothelial carcinoma, CRC colorectal carcinoma, MSI-H high levels of microsatellite instability, MSS microsatellite stable, DCIS ductal carcinoma in situ

**Supplementary Table 5 (1/4): All MSI analyses for individuals with a diagnosis of *MSH2*-LS (cases 2.1-2.5 and case A)**

| **Case ID** | **Sex** | **Sample Type** | **MSI result** | **MSI score** | **Days since primary urine sample** | **Notes on sample timing** |
| --- | --- | --- | --- | --- | --- | --- |
| **2.1** | Female | Primary Urine | MSI-H | 27.36 | 0 |  |
|  |  | Urine | MSI-H | 3.665 | 61 | Initial repeat- delayed due to unrelated illness and admission |
|  |  | Urine | MSI-H | 11.032 | 223 |  |
|  |  | Blood | MSS | -22.322 | 245 |  |
|  |  | Urine | MSI-H | 26.813 | 284 | Prior to first URS with biopsies |
|  |  | Tumour (UTUC) | MSI-H | 17.26 | 284 |  |
|  |  | Urine | MSS | -10.958 | 292 | 1 week after first URS with biopsies |
|  |  | Urine | MSS | -22.311 | 378 | Prior to second URS with laser ablation |
|  |  | Urine | MSS | -22.329 | 389 | Postal sample following second URS with laser ablation |
| **2.2** | Male | Primary Urine | Borderline | -0.58 | 0 |  |
|  |  | Urine | MSI-H | 16.359 | 33 | Initial repeat |
|  |  | Urine | MSI-H | 7.217 | 55 |  |
|  |  | Blood | MSS | -22.38 | 61 |  |
|  |  | Urine | MSI-H | 15.869 | 107 |  |
|  |  | Urine | MSI-H | 29.143 | 129 | Prior to first URS with tumour ablation and removal |
|  |  | Tumour (UTUC) | MSI-H | 14.77 | 159 |  |
|  |  | Urine | MSS | -22.38 | 176 | Follow up sample after first URS |
|  |  | Urine | MSS | -22.052 | 264 | Prior to follow up URS- no disease detected on URS |
|  |  | Urine | MSS | -22.622 | 285 | After follow up URS |
|  |  | Urine | MSS | -22.339 | 401 | Prior to follow up URS- no disease detected on URS |
|  |  | Urine | MSS | -15.592 | 411 | After follow up URS |
|  |  | Urine | MSI-H | 2.042 | 593 | Prior to follow up URS- small recurrence detected. Insufficient tissue to biopsy. Further laser ablation. |
|  |  | Urine | MSS | -22.331 | 604 | After follow up URS |
| **2.3** | Male | Primary Urine | MSI-H | 26.336 | 0 |  |
|  |  | Urine | MSI-H | 26.336 | 34 | Initial repeat |
|  |  | Blood | MSS | -22.598 | 63 |  |
|  |  | Urine | MSI-H | 29.277 | 227 | Prior to nephroureterectomy |
|  |  | Tumour | MSI-H | 23.857 | 320 |  |
| **2.4** | Female | Primary Urine | MSI-H | 10.344 | 0 |  |
|  |  | Urine | MSI-H | 23.851 | 28 | Initial repeat |
|  |  | Blood | MSS | -22.38 | 53 |  |
|  |  | Urine | MSS | -22.38 | 141 | Prior to nephrectomy |
|  |  | Tumour (RCC) | MSS | -22.38 | 141 | All RCC blocks tested: All MSS |
|  |  | Urine | MSI-H | 10.344 | 143 | After nephrectomy (taken during admission) |
|  |  | Urine | MSS | -22.38 | 213 | Repeat due to previous results |
|  |  | Urine | MSS | -22.38 | 253 | Repeat due to previous results |
|  |  | Urine | MSI-H | 18.401 | 318 | Postal sample prior to planned 1st cystoscopy |
|  |  | Tumour (BUC) | MSI-H | 28.946 | 361 |  |
|  |  | Urine | MSS | -22.62 | 364 | Follow-up sample after 1st cystoscopy with TURBT |
|  |  | Urine | MSS | -22.33 | 384 | Prior to 2nd cystoscopy with re-resection of scar tissue. No macroscopic residual disease but biopsy showed focal residual BUC. |
|  |  | Urine | MSS | -22.322 | 442 | Follow up sample after repeat cystoscopy. |
| **2.5** | Female | Primary Urine | MSI-H | 15.15 | 0 |  |
|  |  | Urine | MSI-H | 4.506 | 17 | Initial repeat |
|  |  | Urine | MSS | -19.439 | 39 |  |
|  |  | Blood | MSS | -19.439 | 39 |  |
|  |  | Urine | MSS | -22.38 | 48 |  |
|  |  | 12month+ follow up urine | MSS | -22.37 | 632 | 1+ year follow up for MSS/No cancer cases |
| **A** | Male | Tumour (BUC) | MSI-H | 14.69 | -99 |  |
|  |  | Primary Urine | MSI-H | 27.758 | 0 | Primary urine sample taken 99 days after TURBT for BUC |
|  |  | Urine | MSI-H | 27.758 | 15 | Initial repeat |
|  |  | Urine | MSI-H | 2.896 | 41 | Performed following repeat TURBT. Incomplete resection due to disease around the ureteric opening |
|  |  | Urine | MSI-H | 27.758 | 119 | Persistent disease- not for definitive surgery |
|  |  | Urine | MSI-H | 27.758 | 182 | Persistent disease- not for definitive surgery |

**Supplementary Table 5 (2/4): All MSI analyses for individuals with a diagnosis of *MSH2*-LS (cases 2.6-2.29)**

| **Case ID** | **Sex** | **Sample Type** | **MSI result** | **MSI score** | **Days since primary urine sample** |
| --- | --- | --- | --- | --- | --- |
| Case2.6 | Male | Primary Urine | MSS | -21.849 | 0 |
|  |  | 12month + follow Up urine | MSS | -22.325 | 561 |
| Case2.7 | Male | Primary Urine | MSS | -22.778 | 0 |
|  |  | 12month + follow Up urine | MSS | -14.999 | 561 |
| Case2.8 | Female | Primary Urine | MSS | -23.129 | 0 |
|  |  | 12month + follow Up urine | MSS | -22.637 | 630 |
| Case2.9 | Female | Primary Urine | MSS | -21.849 | 0 |
|  |  | 12month + follow Up urine | MSS | -12.091 | 497 |
| Case2.10 | Female | Primary Urine | MSS | -22.598 | 0 |
|  |  | 12month + follow Up urine | MSS | -22.62 | 571 |
| Case2.11 | Male | Primary Urine | MSS | -21.849 | 0 |
|  |  | 12month + follow Up urine | MSS | -22.334 | 585 |
| Case2.12 | Female | Primary Urine | MSS | -22.598 | 0 |
|  |  | 12month + follow Up urine | MSS | -22.328 | 536 |
| Case2.13 | Female | Primary Urine | MSS | -23.129 | 0 |
|  |  | 12month + follow Up urine | MSS | -22.339 | 583 |
| Case2.14 | Male | Primary Urine | MSS | -22.598 | 0 |
|  |  | 12month + follow Up urine | MSS | -22.347 | 679 |
| Case2.15 | Female | Primary Urine | MSS | -21.849 | 0 |
|  |  | 12month + follow Up urine | MSS | -22.14 | 536 |
| Case2.16 | Female | Primary Urine | MSS | -15.532 | 0 |
|  |  | 12month + follow Up urine | MSS | -22.124 | 729 |
| Case2.17 | Female | Primary Urine | MSS | -21.849 | 0 |
|  |  | 12month + follow Up urine | MSS | -22.122 | 547 |
| Case2.18 | Female | Primary Urine | MSS | -17.858 | 0 |
|  |  | 12month + follow Up urine | MSS | -18.327 | 581 |
| Case2.19 | Female | Primary Urine | MSS | -22.598 | 0 |
|  |  | 12month + follow Up urine | MSS | -22.354 | 521 |
| Case2.20 | Male | Primary Urine | MSS | -22.38 | 0 |
|  |  | 12month + follow Up urine | MSS | -22.636 | 613 |
| Case2.21 | Male | Primary Urine | MSS | -18.912 | 0 |
|  |  | 12month + follow Up urine | MSS | -22.323 | 521 |
| Case2.22 | Female | Primary Urine | MSS | -23.129 | 0 |
|  |  | 12month + follow Up urine | MSS | -19.259 | 611 |
| Case2.23 | Female | Primary Urine | MSS | -23.129 | 0 |
| Case2.24 | Male | Primary Urine | MSS | -19.753 | 0 |
|  |  | 12month + follow Up urine | MSS | -18.947 | 583 |
| Case2.25 | Male | Primary Urine | MSS | -14.482 | 0 |
|  |  | 12month + follow Up urine | MSS | -18.678 | 590 |
| Case2.26 | Female | Primary Urine | MSS | -15.657 | 0 |
|  |  | 12month + follow Up urine | MSS | -19.256 | 521 |
| Case2.27 | Female | Primary Urine | MSS | -21.849 | 0 |
|  |  | 12month + follow Up urine | MSS | -22.049 | 516 |
| Case2.28 | Female | Primary Urine | MSS | -21.849 | 0 |
|  |  | 12month + follow Up urine | MSS | -22.34 | 490 |
| Case2.29 | Female | Primary Urine | MSS | -21.849 | 0 |
|  |  | 12month + follow Up urine | MSS | -18.628 | 527 |

| **Case ID** | **Sex** | **Sample Type** | **MSI result** | **MSI score** | **Days since primary urine sample** |
| --- | --- | --- | --- | --- | --- |
| Case2.30 | Female | Primary Urine | MSS | -21.849 | 0 |
|  |  | 12month + follow Up urine | MSS | -22.354 | 555 |
| Case2.31 | Male | Primary Urine | MSS | -23.129 | 0 |
|  |  | 12month + follow Up urine | MSS | -18.639 | 520 |
| Case2.32 | Male | Primary Urine | MSS | -22.38 | 0 |
|  |  | 12month + follow Up urine | MSS | -22.615 | 641 |
| Case2.33 | Male | Primary Urine | MSS | -19.005 | 0 |
|  |  | 12month + follow Up urine | MSS | -15.272 | 497 |
| Case2.34 | Female | Primary Urine | MSS | -22.598 | 0 |
| Case2.35 | Female | Primary Urine | MSS | -23.129 | 0 |
|  |  | 12month + follow Up urine | MSS | -22.606 | 568 |
| Case2.36 | Male | Primary Urine | MSS | -23.129 | 0 |
|  |  | 12month + follow Up urine | MSS | -22.325 | 526 |
| Case2.37 | Female | Primary Urine | MSS | -21.849 | 0 |
|  |  | 12month + follow Up urine | MSS | -22.331 | 508 |
| Case2.38 | Male | Primary Urine | MSS | -19.005 | 0 |
|  |  | 12month + follow Up urine | MSS | -14.63 | 780 |
| Case2.39 | Female | Primary Urine | MSS | -22.996 | 0 |
|  |  | 12month + follow Up urine | MSS | -22.619 | 667 |
| Case2.40 | Female | Primary Urine | MSS | -22.247 | 0 |
|  |  | 12month + follow Up urine | MSS | -22.324 | 665 |
| Case2.41 | Female | Primary Urine | MSS | -21.849 | 0 |
|  |  | 12month + follow Up urine | MSS | -18.184 | 633 |
| Case2.42 | Male | Primary Urine | MSS | -23.129 | 0 |
|  |  | 12month + follow Up urine | MSS | -22.327 | 514 |
| Case2.43 | Female | Primary Urine | MSS | -22.778 | 0 |
|  |  | 12month + follow Up urine | MSS | -22.618 | 577 |
| Case2.44 | Male | Primary Urine | MSS | -22.38 | 0 |
|  |  | 12month + follow Up urine | MSS | -22.317 | 518 |
| Case2.45 | Female | Primary Urine | MSS | -23.129 | 0 |
|  |  | 12month + follow Up urine | MSS | -22.594 | 597 |
| Case2.46 | Female | Primary Urine | MSS | -21.849 | 0 |
| Case2.47 | Male | Primary Urine | MSS | -22.598 | 0 |
|  |  | 12month + follow Up urine | MSS | -22.323 | 493 |
| Case2.48 | Female | Primary Urine | MSS | -22.38 | 0 |
| Case2.49 | Male | Primary Urine | MSS | -22.38 | 0 |
| Case2.50 | Male | Primary Urine | MSS | -22.38 | 0 |
|  |  | 12month + follow Up urine | MSS | -22.615 | 639 |
| Case2.51 | Male | Primary Urine | MSS | -21.849 | 0 |
|  |  | 12month + follow Up urine | MSS | -22.074 | 639 |
| Case2.52 | Male | Primary Urine | MSS | -23.129 | 0 |
|  |  | 12month + follow Up urine | MSS | -22.316 | 530 |
| Case2.53 | Male | Primary Urine | MSS | -17.858 | 0 |
|  |  | 12month + follow Up urine | MSS | -22.605 | 491 |
| Case2.54 | Male | Primary Urine | MSS | -21.849 | 0 |
|  |  | 12month + follow Up urine | MSS | -22.325 | 539 |
| Case2.55 | Male | Primary Urine | MSS | -23.129 | 0 |
|  |  | 12month + follow Up urine | MSS | -22.339 | 641 |
| Case2.56 | Female | Primary Urine | MSS | -23.129 | 0 |
|  |  | 12month + follow Up urine | MSS | -22.312 | 633 |
| Case2.57 | Female | Primary Urine | MSS | -22.38 | 0 |
|  |  | 12month + follow Up urine | MSS | -22.327 | 695 |
| Case2.58 | Female | Primary Urine | MSS | -23.527 | 0 |
|  |  | 12month + follow Up urine | MSS | -22.329 | 633 |

**Supplementary Table 5 (3/4): All MSI analyses for individuals with a diagnosis of *MSH2*-LS (cases 2.30-2.58)**

**Supplementary Table 5 (4/4): All MSI analyses for individuals with a diagnosis of *MSH2*-LS (cases 2.59-2.80)**

| **Case ID** | **Sex** | **Sample Type** | **MSI result** | **MSI score** | **Days since primary urine sample** |
| --- | --- | --- | --- | --- | --- |
| Case2.59 | Male | Primary Urine | MSS | -15.958 | 0 |
|  |  | 12month + follow Up urine | MSS | -22.072 | 619 |
| Case2.60 | Male | Primary Urine | MSS | -21.849 | 0 |
|  |  | 12month + follow Up urine | MSS | -22.315 | 506 |
| Case2.61 | Male | Primary Urine | MSS | -21.849 | 0 |
|  |  | 12month + follow Up urine | MSS | -19.671 | 513 |
| Case2.62 | Male | Primary Urine | MSS | -18.787 | 0 |
|  |  | 12month + follow Up urine | MSS | -22.355 | 609 |
| Case2.63 | Male | Primary Urine | MSS | -21.849 | 0 |
|  |  | 12month + follow Up urine | MSS | -22.603 | 605 |
| Case2.64 | Female | Primary Urine | MSS | -22.598 | 0 |
|  |  | 12month + follow Up urine | MSS | -22.318 | 535 |
| Case2.65 | Male | Primary Urine | MSS | -22.598 | 0 |
|  |  | 12month + follow Up urine | MSS | -18.436 | 553 |
| Case2.66 | Female | Primary Urine | MSS | -22.778 | 0 |
|  |  | 12month + follow Up urine | MSS | -22.613 | 517 |
| Case2.67 | Female | Primary Urine | MSS | -19.005 | 0 |
|  |  | 12month + follow Up urine | MSS | -22.077 | 583 |
| Case2.68 | Female | Primary Urine | MSS | -20.179 | 0 |
|  |  | 12month + follow Up urine | MSS | -22.336 | 647 |
| Case2.69 | Male | Primary Urine | MSS | -21.849 | 0 |
|  |  | 12month + follow Up urine | MSS | -18.372 | 577 |
| Case2.70 | Male | Primary Urine | MSS | -22.598 | 0 |
| Case2.71 | Female | Primary Urine | MSS | -22.598 | 0 |
|  |  | 12month + follow Up urine | MSS | -22.602 | 497 |
| Case2.72 | Male | Primary Urine | MSS | -22.598 | 0 |
|  |  | 12month + follow Up urine | MSS | -22.616 | 610 |
| Case2.73 | Female | Primary Urine | MSS | -17.858 | 0 |
|  |  | 12month + follow Up urine | MSS | -18.325 | 603 |
| Case2.74 | Female | Primary Urine | MSS | -22.38 | 0 |
|  |  | 12month + follow Up urine | MSS | -22.06 | 484 |
| Case2.75 | Male | Primary Urine | MSS | -21.849 | 0 |
|  |  | 12month + follow Up urine | MSS | -22.343 | 508 |
| Case2.76 | Female | Primary Urine | MSS | -23.527 | 0 |
|  |  | 12month + follow Up urine | MSS | -22.324 | 591 |
| Case2.77 | Female | Primary Urine | MSS | -22.598 | 0 |
|  |  | 12month + follow Up urine | MSS | -22.349 | 497 |
| Case2.78 | Male | Primary Urine | MSS | -23.129 | 0 |
|  |  | 12month + follow Up urine | MSS | -22.111 | 772 |
| Case2.79 | Female | Primary Urine | MSS | -15.709 | 0 |
|  |  | 12month + follow Up urine | MSS | -22.334 | 647 |
| Case2.80 | Female | Primary Urine | MSS | -23.129 | 0 |
|  |  | 12month + follow Up urine | MSS | -22.616 | 585 |

**Supplementary Table 5: All MSI analyses for individuals with a diagnosis of *MSH2*-LS**

Cases are numbered from case 2.1 to case 2.80 and case A, with the six cases with MSI-High results of primary urine sample analyses being cases 2.1-2.5 and case A. Full details of the interpretation of MSI scores is provided in the main methodology: MSI scores within the borderline range of -5 to +5 were repeated where available, or considered MSI-High where persistently within this range or a repeat was not possible. The date of the primary urine sample analysis was denoted as day 0 in all cases, with all other dates calculated from this time.
Abbreviations: UTUC Upper tract urothelial carcinoma, RCC Renal cell carcinoma, BUC Bladder urothelial carcinoma, MSI-H High levels of microsatellite instability, MSS microsatellite stable, URS Ureterorenoscopy, TURBT Transurethral resection of bladder tumour

**Supplementary Table 6: Cross tabulation of the index test (MSI testing) with the reference standard(s)**

| **Unselected UTUC cases (n=50)** | | | |
| --- | --- | --- | --- |
| **Tumour MSI testing with the Newcastle MSI-Plus assay vs Tumour MMR IHC** | | | |
|  | **MMRd on MMR IHC** | **MMRp on MMR IHC** | **Total(s)** |
| **Tumour MSI-High** | 3 | 0 | 3 |
| **Tumour MSS** | 0 | 46 | 46 |
| **Total(s)** | 3 | 46 | 49 |
| **Pre-operative urine MSI testing vs tumour MSI testing using the Newcastle MSI-Plus assay** | | | |
|  | **Tumour MSI-High** | **Tumour MSS** | **Total(s)** |
| **Urine MSI-High** | 3* | 0 | 3 |
| **Urine MSS** | 0 | 47 | 47 |
| **Total(s)** | 3 | 47 | 50 |
| **Asymptomatic individuals with a diagnosis of *MSH2*-LS (n=80)** | | | |
|  | **Urothelial carcinoma present (Histology)** | **No cancer diagnosis after 12m follow up** | **Total(s)** |
| **Urine MSI-High** | 4* | 1 | 5 |
| **Urine MSS** | 0 | 75 | 75 |
| **Total(s)** | 4 | 76 | 80 |

**Supplementary Table 6: Cross tabulation of the index test (MSI testing) with the reference standard(s)**For cohort 1 (unselected cases of UTUC undergoing nephroureterectomy, n=50), MMR IHC was first used as the reference standard for tumour MSI testing using the Newcastle MSI-Plus assay for the 49 cases where both techniques were performed. Tumour MSI testing was subsequently used as the reference standard for urine MSI testing for all 50 cases.

* Denotes the inclusion of cases where the initial urine MSI result fell within the borderline range of -5 to +5 in the MSI-H results. In the UTUC cohort, repeat pre-operative urine sample collection was not possible for case 1.3 and the borderline result was therefore treated as MSI-H as per our methodology. In the LS cohort, repeat sample collection for case 2.2 gave an MSI-H result.

Abbreviations: UTUC Upper tract urothelial carcinoma, MSI microsatellite instability, MMR IHC mismatch repair protein immunohistochemistry, MMRd mismatch repair deficiency, MMRp mismatch repair proficiency, MSI-H High levels of microsatellite instability, MSS microsatellite stable, LS Lynch syndrome

**Supplementary Table 7: Ultrasound results for individuals with a diagnosis of *MSH2*-LS seen in clinic**

| **Ultrasound result** | **Number of cases (n=52)** |
| --- | --- |
| **No abnormality detected** | 34 |
| **Single renal angiomyolipoma** | 2 |
| **Renal cyst(s)** | 6 (3 simple, 2 complex, 1 combination) |
| **Ovarian cyst** | 1 |
| **Renal mass (RCC)** | 1 |
| **Ultrasound not performed due to recent imaging** | 7 |
| **Did not attend ultrasound appointment** | 1 |

**Supplementary Table 7: Ultrasound results for individuals with a diagnosis of *MSH2*-LS**An ultrasound was offered to all patients seen in the pilot urology genetics combined clinic who had not had urinary tract imaging performed within the previous 6 months and was subsequently performed for 44/52 patients.

Abbreviations: RCC renal cell carcinoma
