# Supplementary material for: Detection of urothelial carcinoma in Lynch syndrome using microsatellite instability analysis of urine cell-free DNA
Source: eBioMedicine. 2025 Oct 25;121:105969. doi: 10.1016/j.ebiom.2025.105969 (PMC12595274; doi:10.1016/j.ebiom.2025.105969)
Supplement: Supplementary Figures [file mmc2.pptx]

## Slide 1
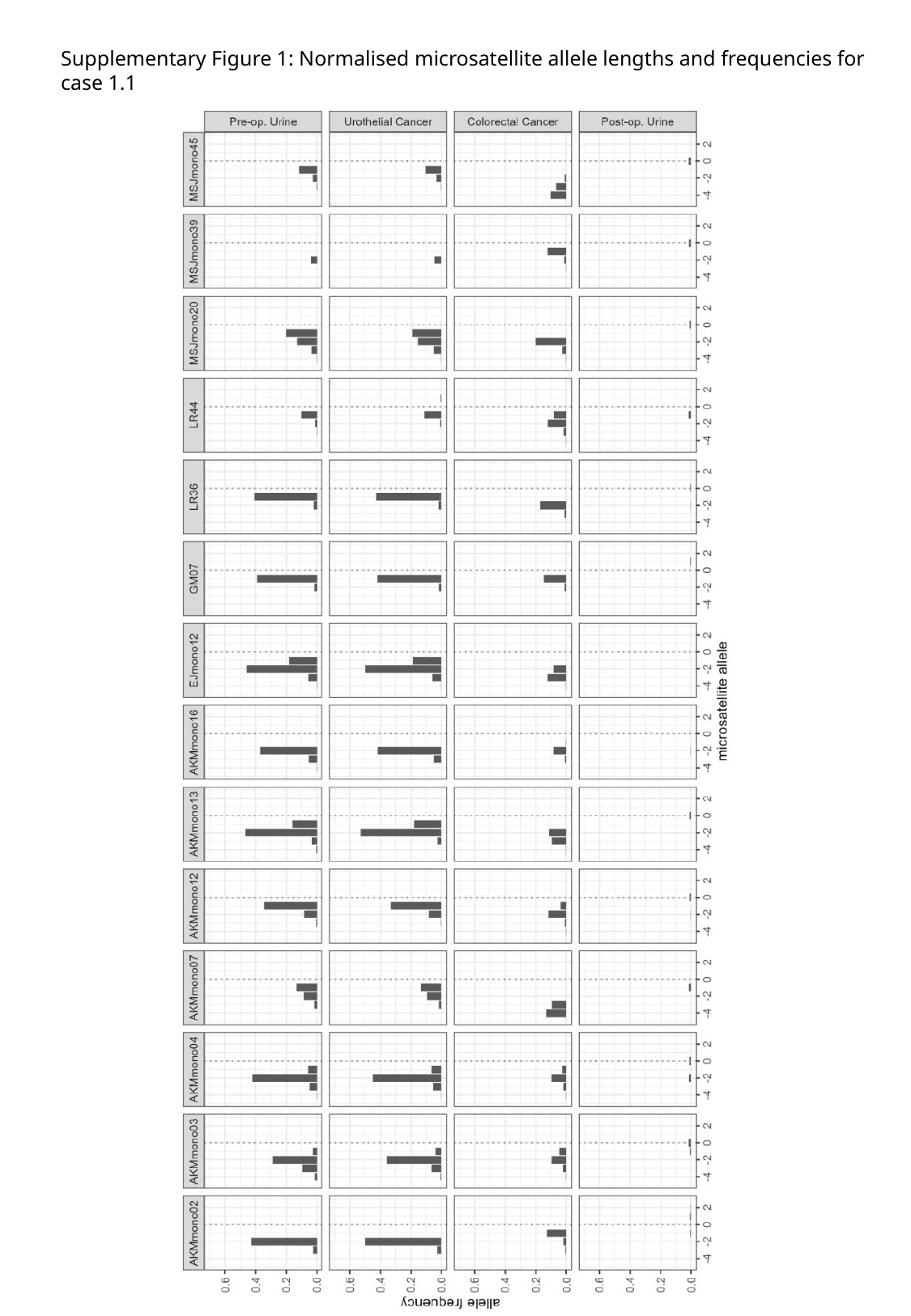

Supplementary Figure 1: Normalised microsatellite allele lengths and frequencies for case 1.1

## Slide 2
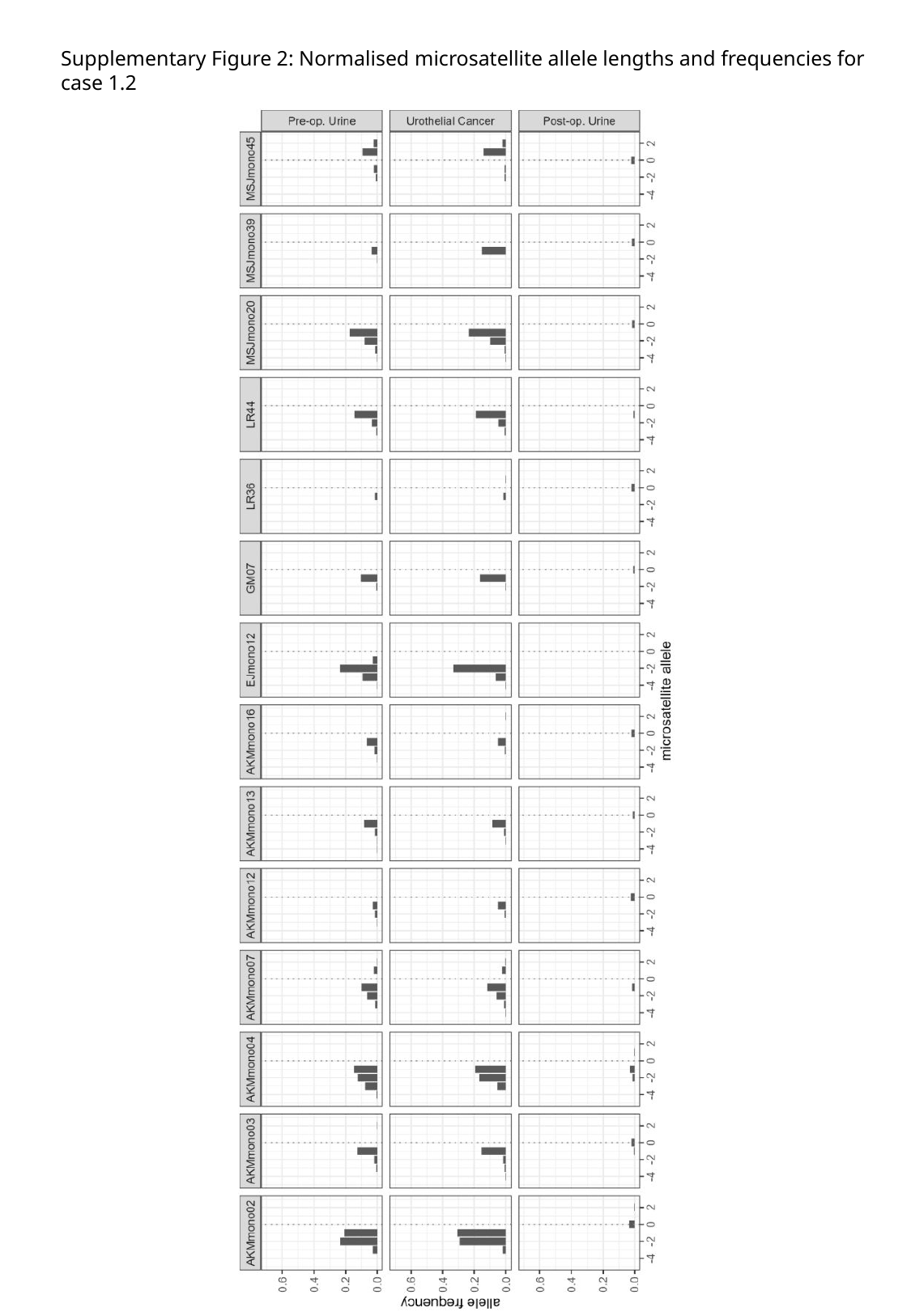

Supplementary Figure 2: Normalised microsatellite allele lengths and frequencies for case 1.2

## Slide 3
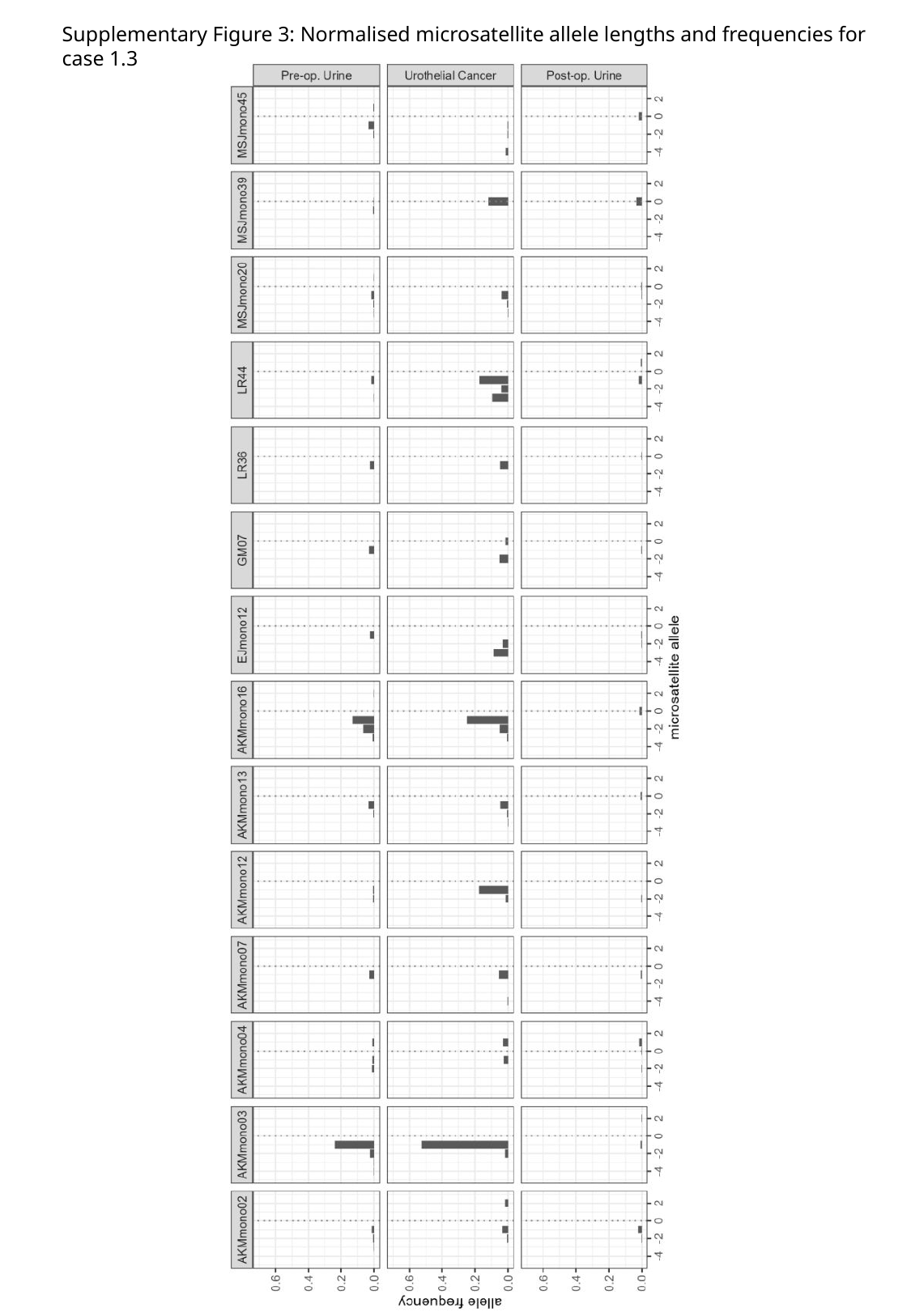

Supplementary Figure 3: Normalised microsatellite allele lengths and frequencies for case 1.3

## Slide 4
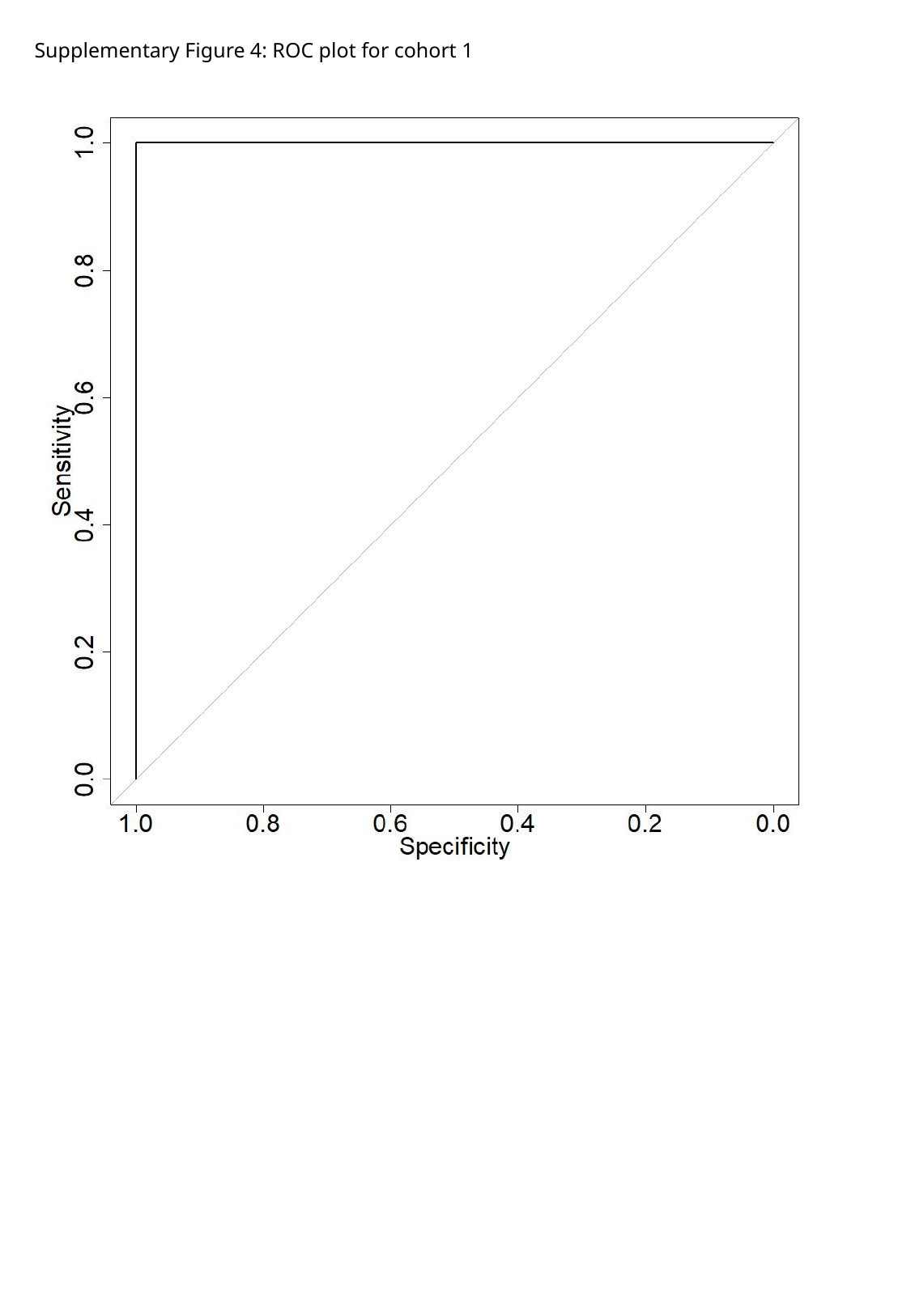

Supplementary Figure 4: ROC plot for cohort 1

## Slide 5
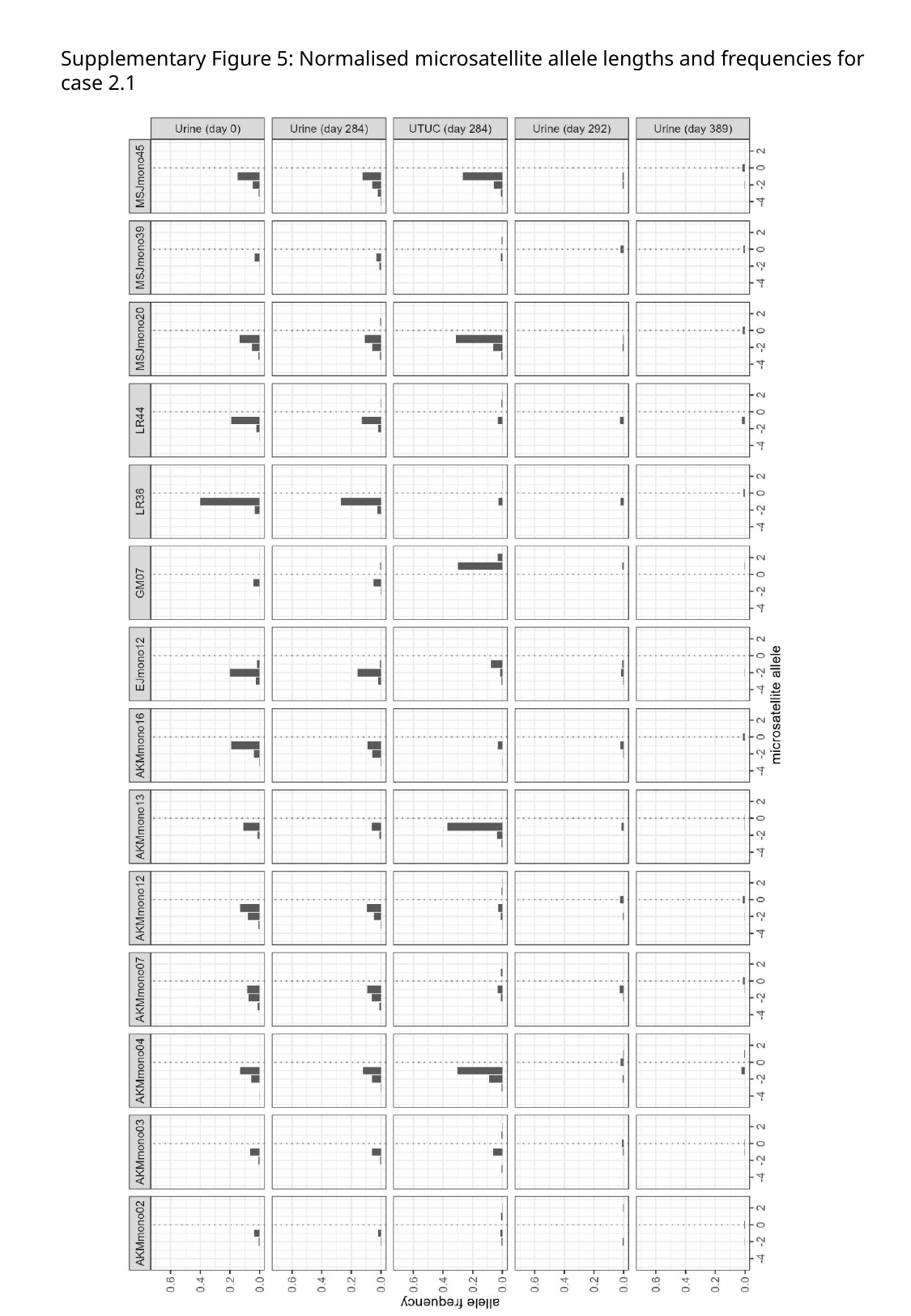

Supplementary Figure 5: Normalised microsatellite allele lengths and frequencies for case 2.1

## Slide 6
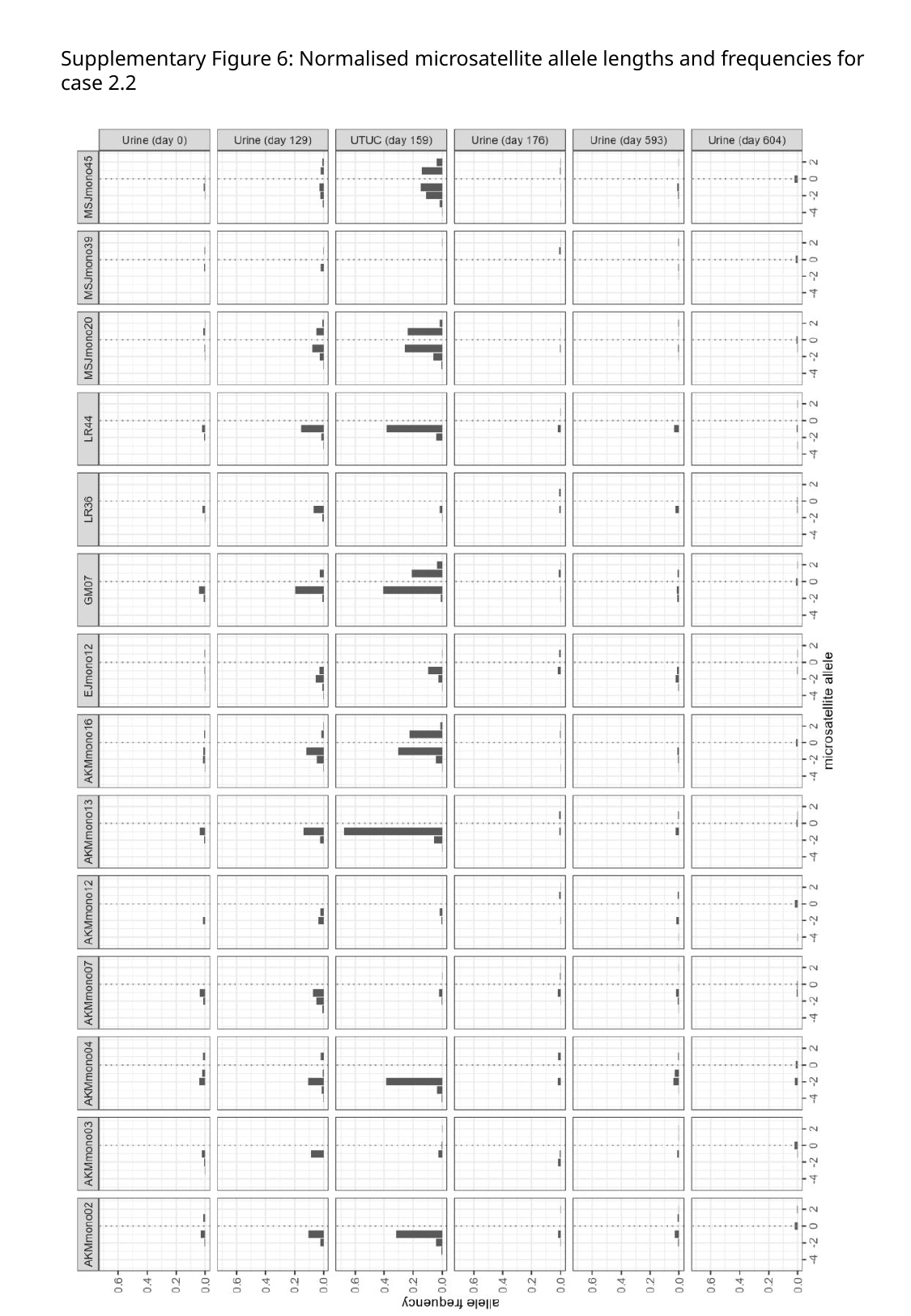

Supplementary Figure 6: Normalised microsatellite allele lengths and frequencies for case 2.2

## Slide 7
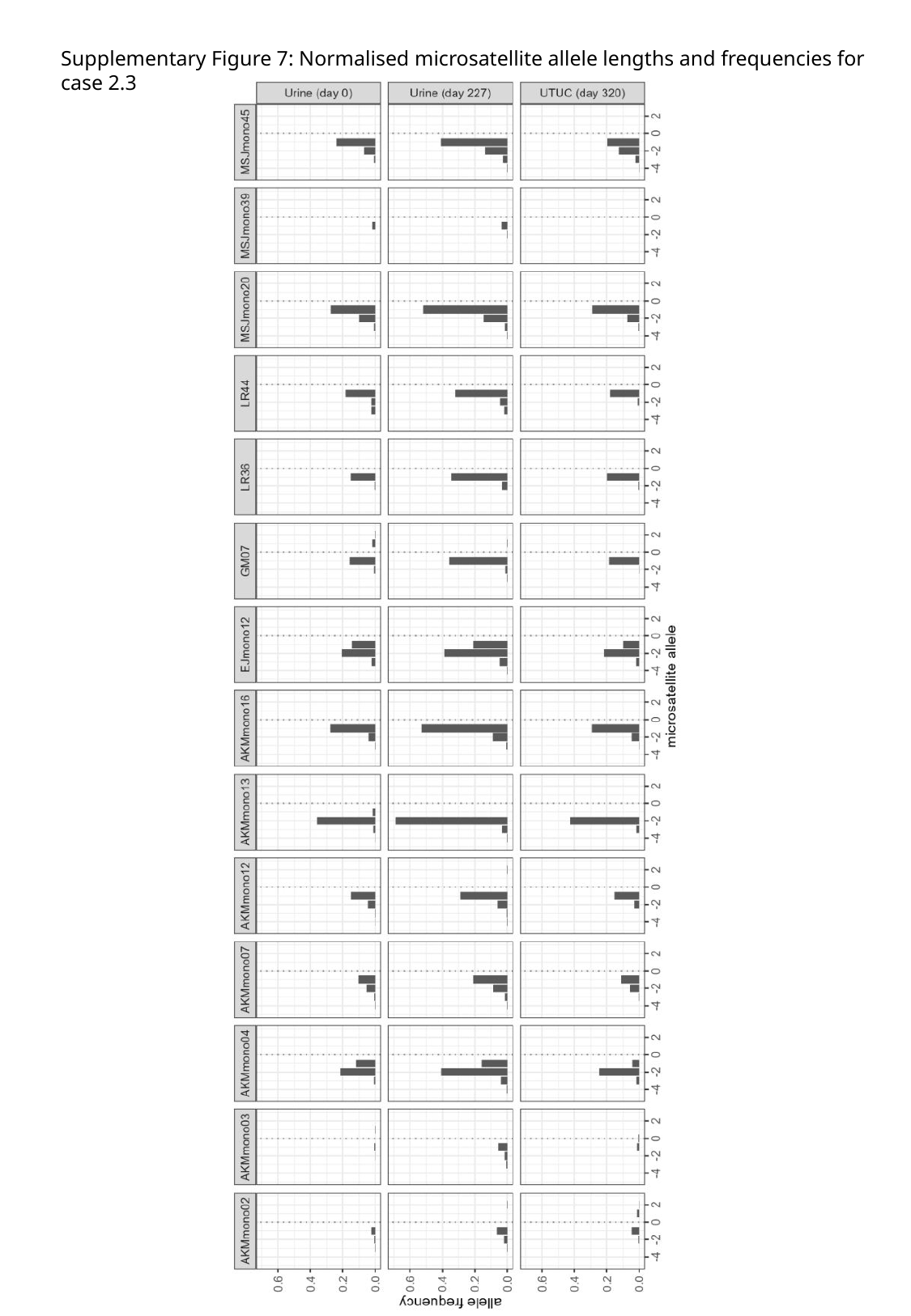

Supplementary Figure 7: Normalised microsatellite allele lengths and frequencies for case 2.3

## Slide 8
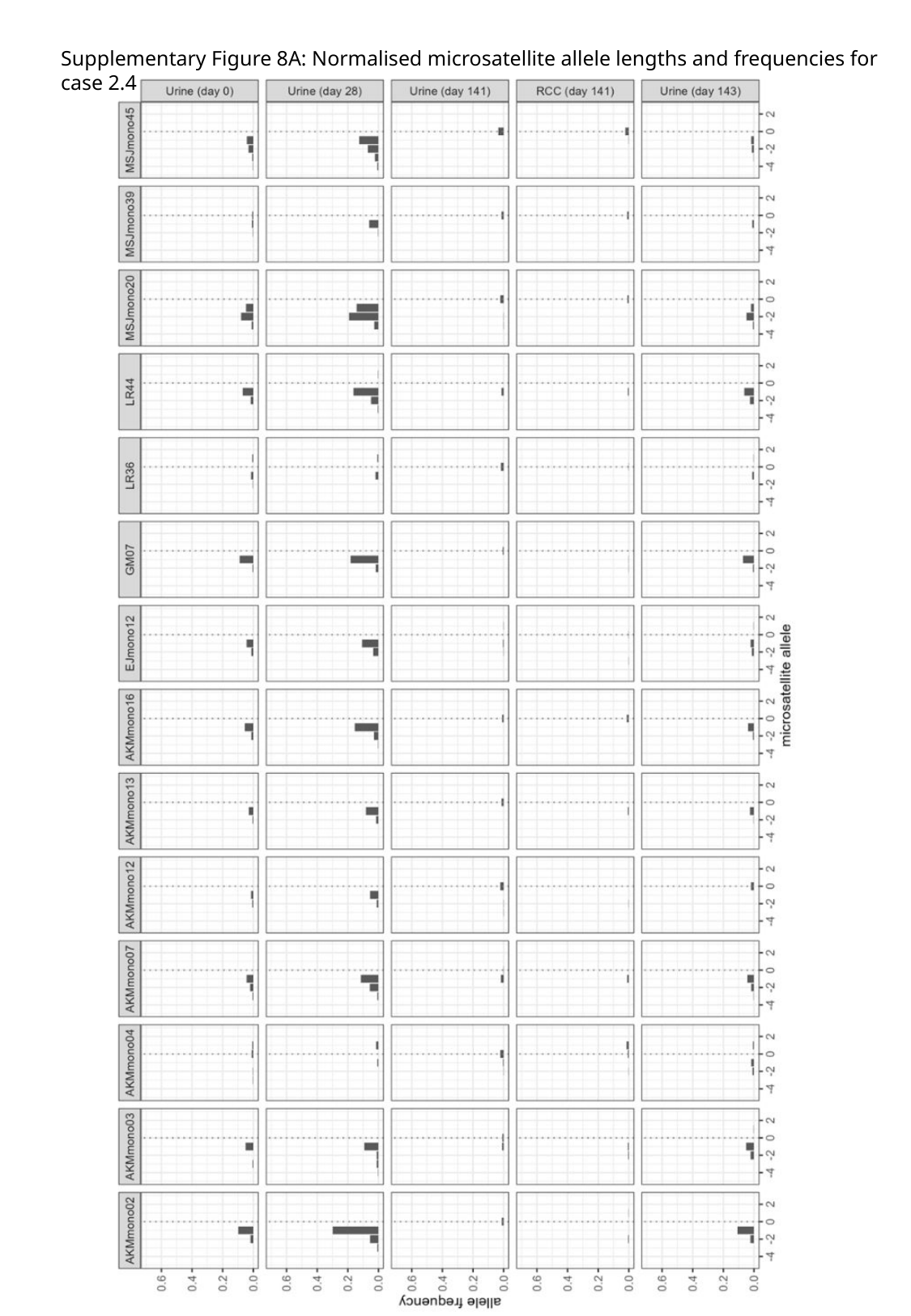

Supplementary Figure 8A: Normalised microsatellite allele lengths and frequencies for case 2.4

## Slide 9
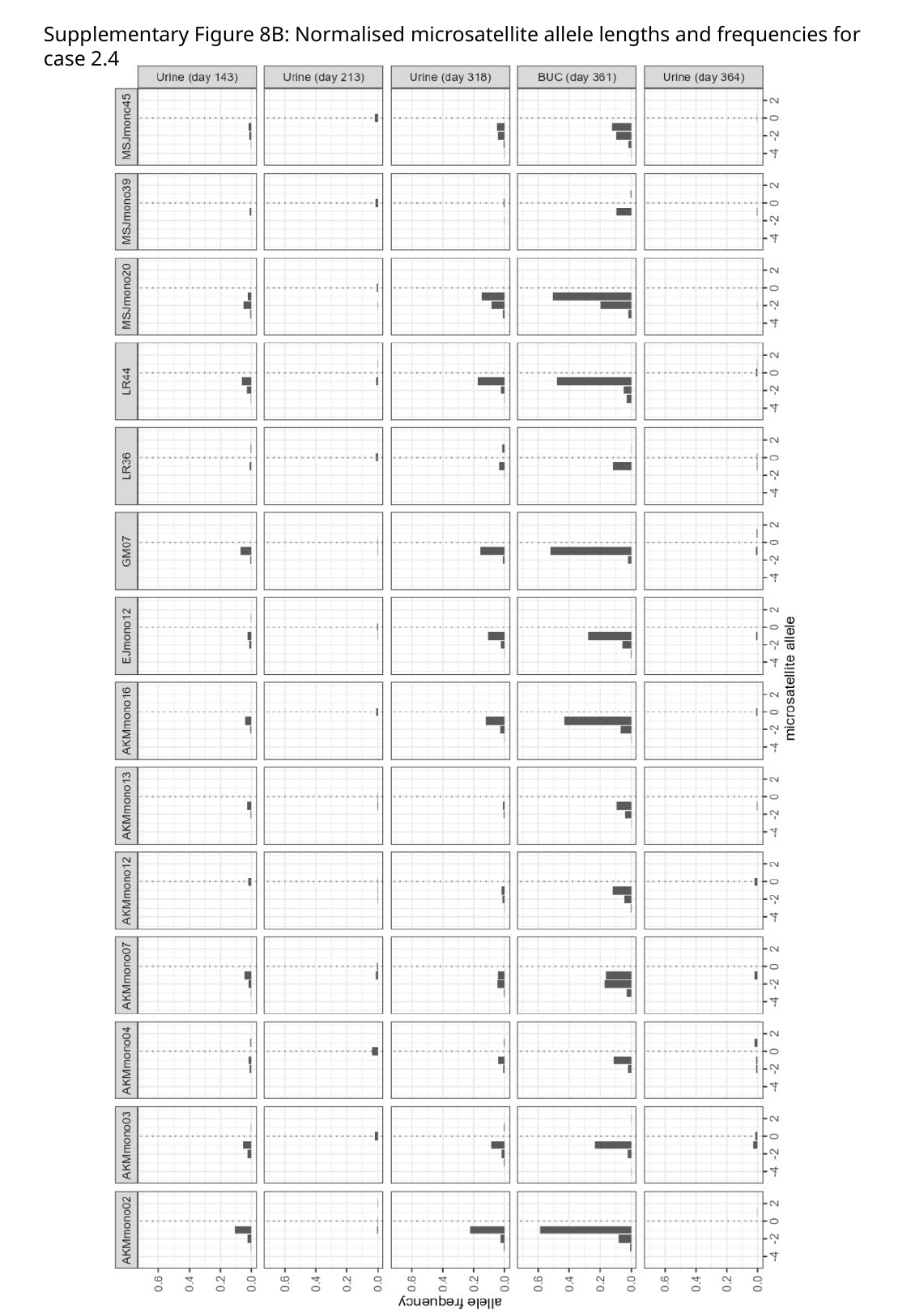

Supplementary Figure 8B: Normalised microsatellite allele lengths and frequencies for case 2.4

## Slide 10
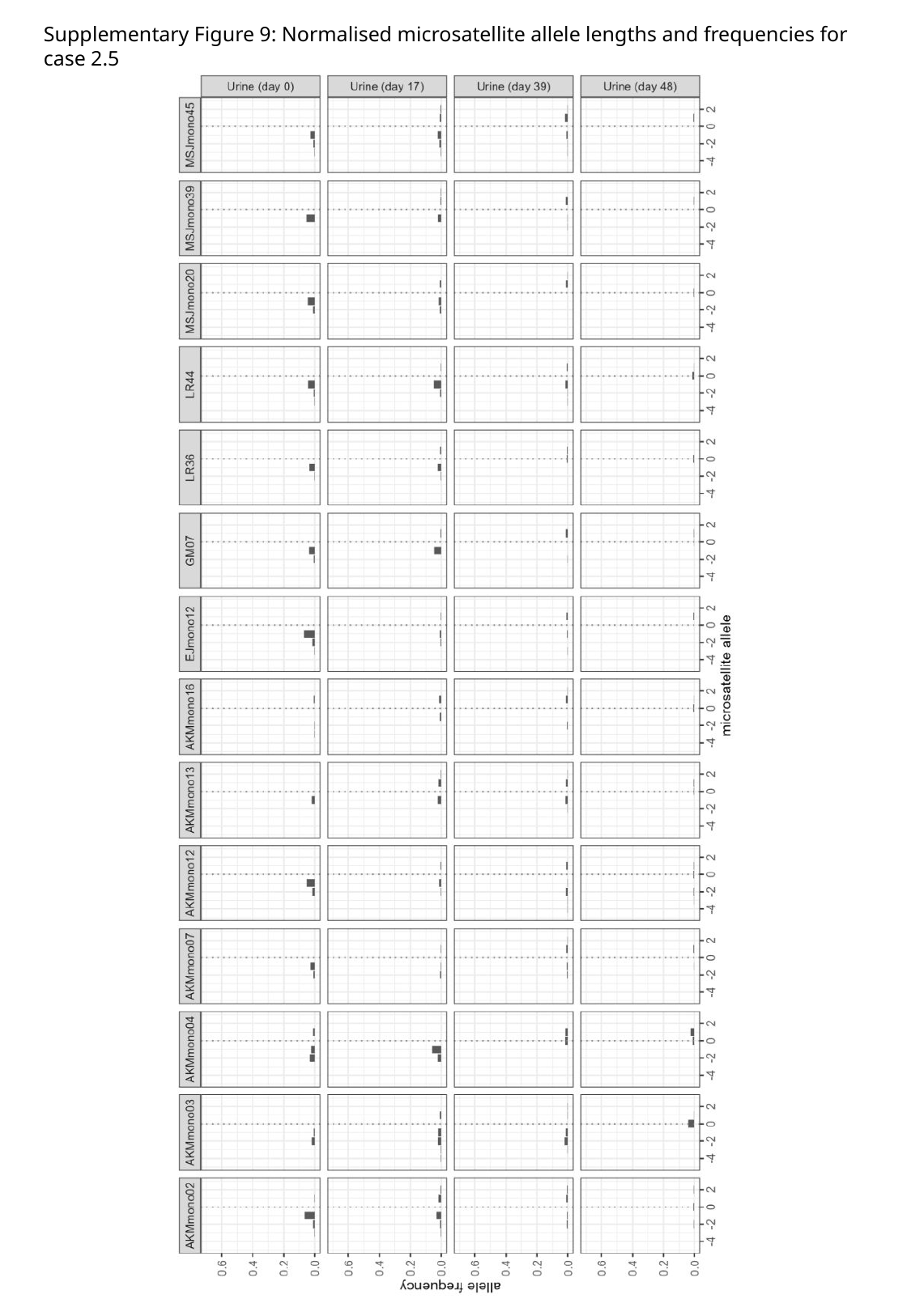

Supplementary Figure 9: Normalised microsatellite allele lengths and frequencies for case 2.5

## Slide 11
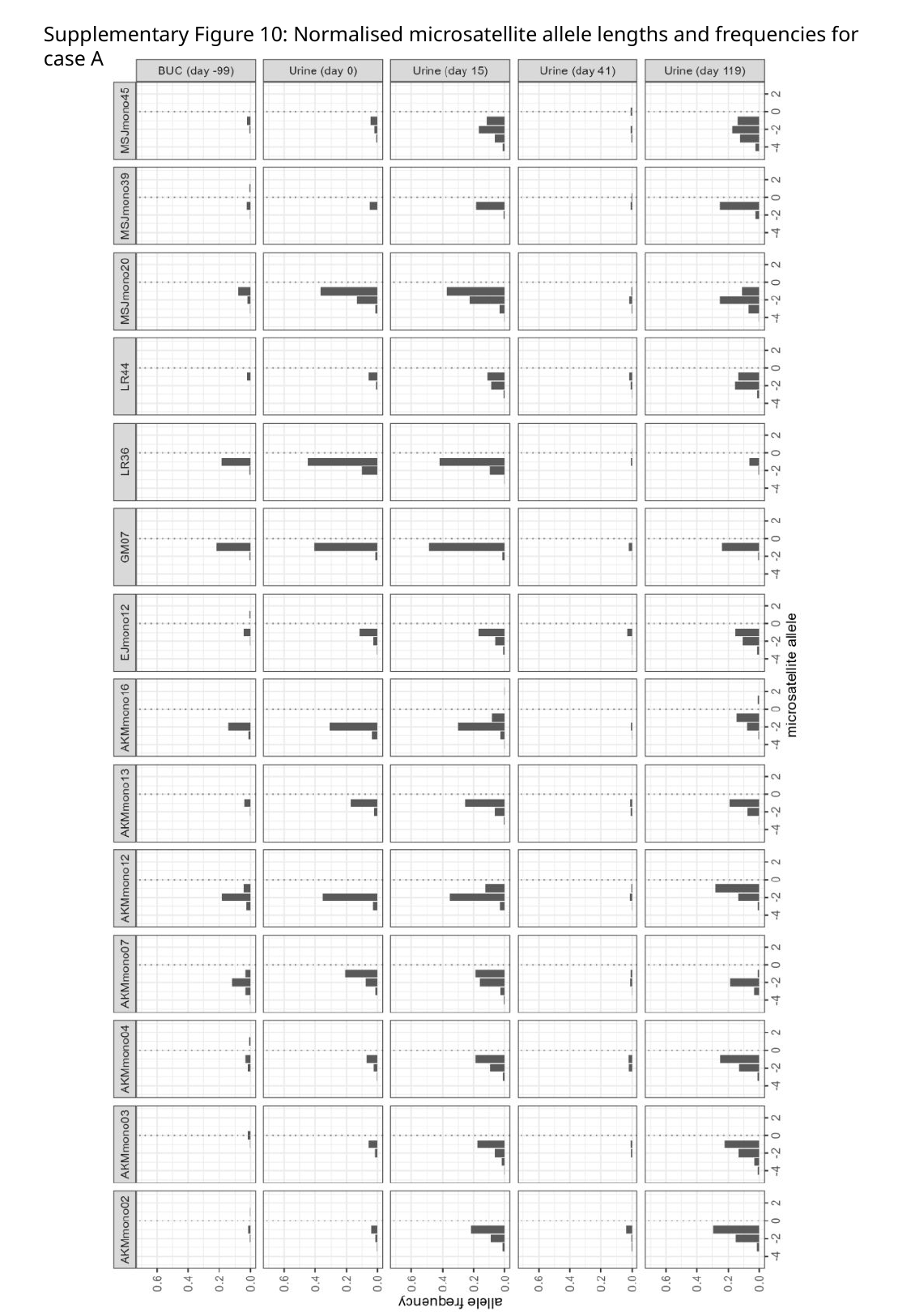

Supplementary Figure 10: Normalised microsatellite allele lengths and frequencies for case A

## Slide 12
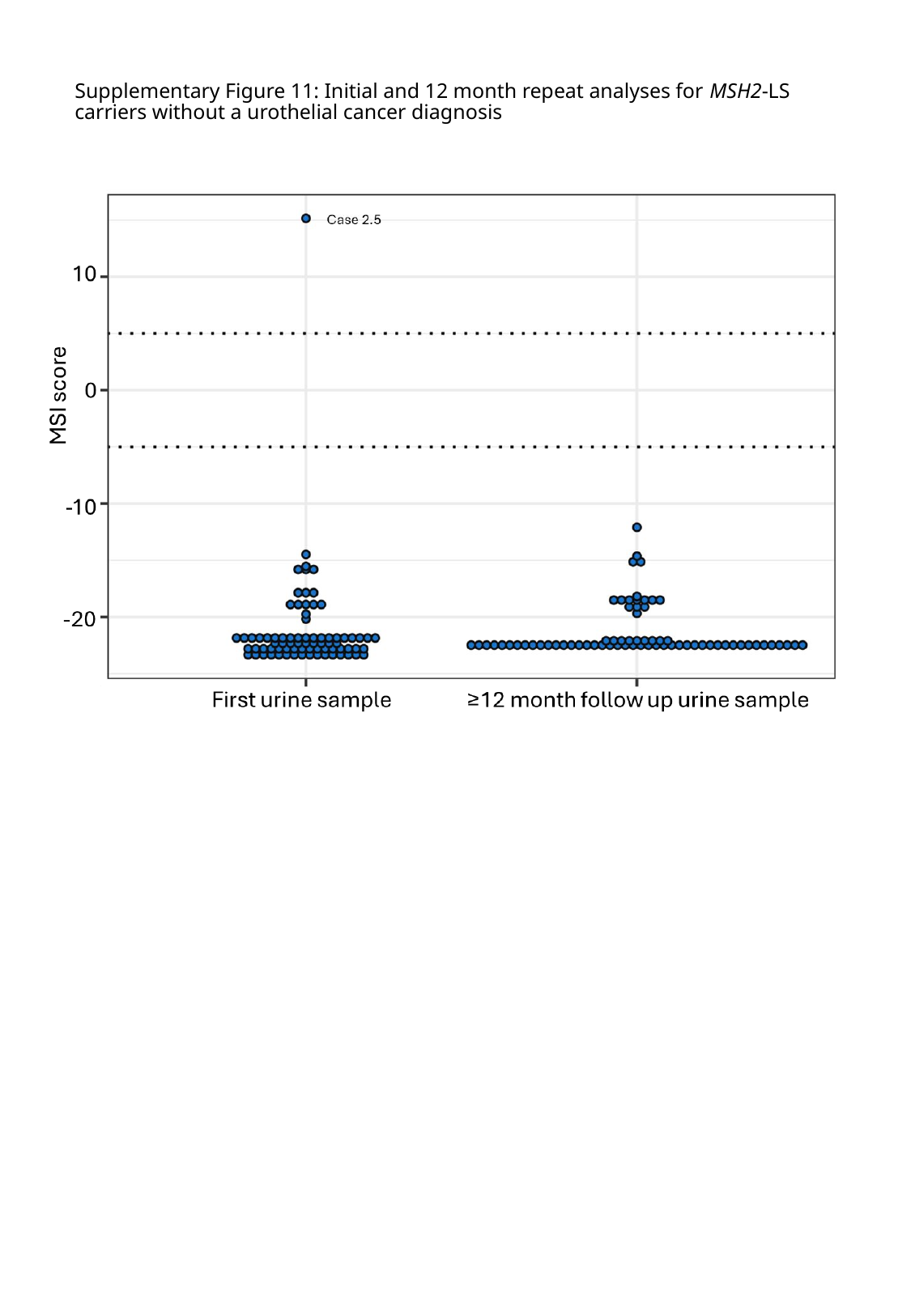

# Supplementary Figure 11: Initial and 12 month repeat analyses for MSH2-LS carriers without a urothelial cancer diagnosis

## Slide 13
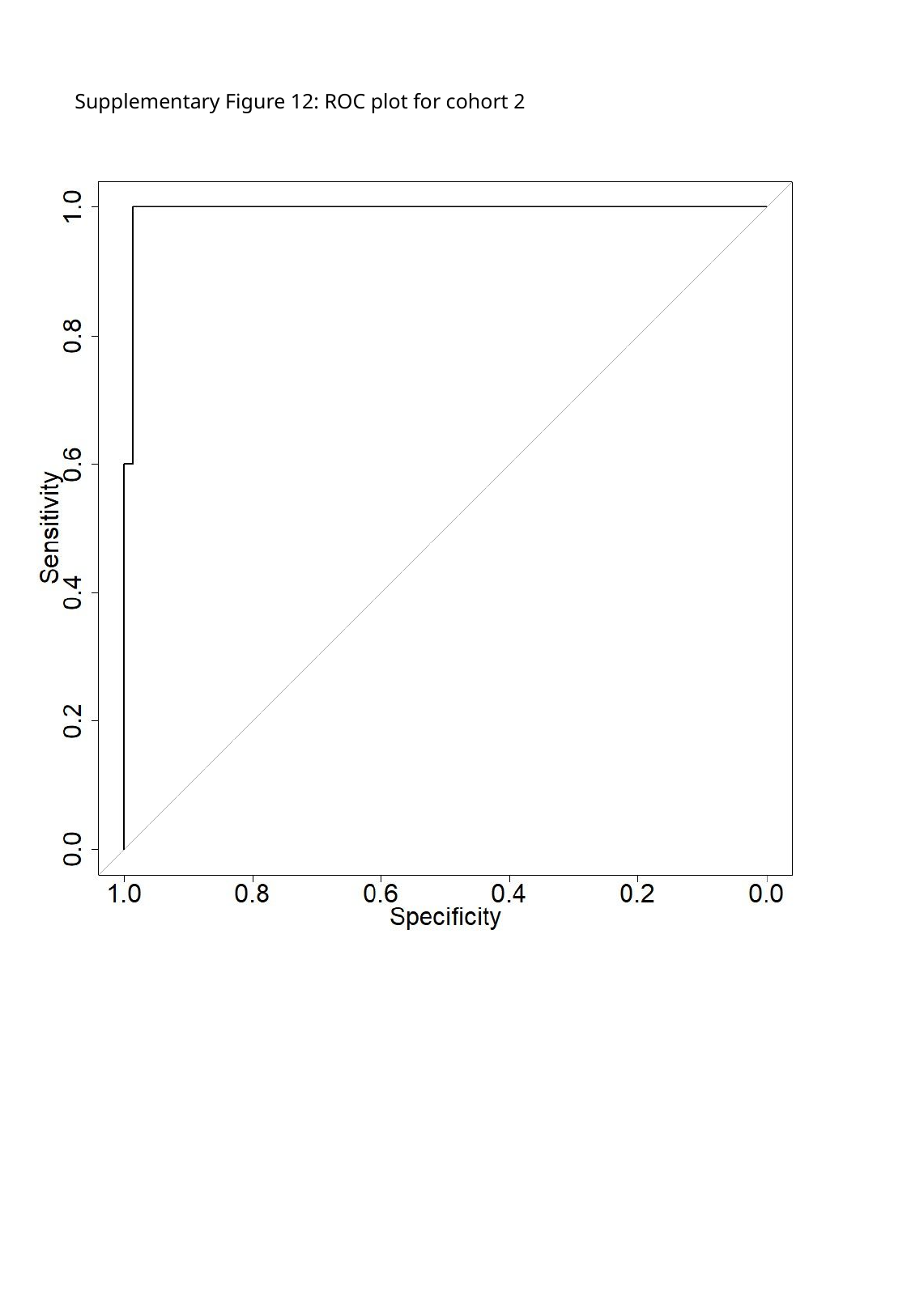

Supplementary Figure 12: ROC plot for cohort 2
